# Piezoelectric Heterojunction-driven Biomedical Revolution: From Construction Principles to Diagnostic and Therapeutic Applications

**DOI:** 10.7150/thno.126489

**Published:** 2026-01-01

**Authors:** Zhiguang Chen, Wei Zhang, Xiujuan Qu, Yunfei Zhang

**Affiliations:** 1 Department of Ultrasound, The First Hospital of China Medical University, China.; 2 Department of Ultrasound, Beijing Tiantan Hospital, Capital Medical University, China.; 3 Department of Medical Oncology, the First Hospital of China Medical University, Shenyang, Liaoning Province 110001, China; Provincial key Laboratory of Anticancer Drugs and Biotherapy of Liaoning Province, the First Hospital of China Medical University, Shenyang, Liaoning Province 110001, China; Clinical Cancer Research Center of Shenyang, the First Hospital of China Medical University, Shenyang, Liaoning Province 110001, China.

**Keywords:** piezoelectric heterojunction, biomedicine, piezoelectric immunotherapy, piezocatalytic medicine

## Abstract

Piezoelectric heterojunctions are emerging as a transformative class of smart biomaterials, revolutionizing biomedical applications through their unique mechano-electrical coupling effects. A central challenge hindering their full potential lies in the systematic understanding and utilization of their complex interfacial enhancement mechanisms. This review aims to establish a comprehensive framework that bridges fundamental principles to clinical translation. We begin with an in-depth analysis of the core enhancement mechanisms in piezoelectric heterojunctions, focusing on the synergistic interplay between the built-in electric field and band engineering that promotes efficient charge separation. We then provide a critical discussion of the ongoing debate surrounding their catalytic mechanism, reconciling the distinctions and connections between band theory and the surface screening charge model. Furthermore, we construct a multidimensional classification system centered on material dimensionality and composition to offer systematic guidance for the rational design and performance optimization of piezoelectric heterojunctions. In terms of applications, this review offers a comprehensive survey of the cutting-edge progress of piezoelectric heterojunctions in efficient cancer therapy, tissue regeneration, antibacterial strategies, and self-powered biosensing. We emphasize that the superiority of these heterojunctions stems from their ability to overcome the bottlenecks of low efficiency and mono-functionality inherent in single-component piezoelectric materials through sophisticated interface design, while also maximizing therapeutic outcomes via multimodal synergistic strategies. Finally, we critically analyze the formidable challenges this field faces concerning biosafety, scalable fabrication, and clinical translation, and offer perspectives on its future development toward intelligent theranostic systems. This review is intended to provide solid theoretical guidance and forward-looking insights for the design of next-generation, high-performance piezoelectric biomaterials.

## Introduction

Since its discovery in α-quartz crystals by the brothers Jacques and Pierre Curie in 1880 1, the piezoelectric effect has evolved from a fundamental physical phenomenon into a key driver of interdisciplinary research. The uniqueness of piezoelectric materials lies in their non-centrosymmetric crystal structure, which enables the bidirectional conversion between mechanical and electrical energy 2. This property has led to their widespread application in traditional fields such as sensors 3, 4, actuators 4, and energy harvesters 5. In recent years, with the rapid advancement of nanoscience and materials science, research on piezoelectric materials has achieved breakthrough progress in the biomedical field 6, ushering in the era of "Piezocatalytic medicine" (PCM). Its core principle involves using the charge carriers generated by piezoelectric materials under mechanical stress to catalyze redox reactions, producing Reactive oxygen species (ROS) for applications such as disease treatment, tissue repair, and antibacterial therapies 6.

However, first-generation piezocatalysts, primarily based on single-component materials, quickly encountered multiple bottlenecks. First, many high-performance inorganic piezoelectric materials (e.g., PZT) contain lead, posing potential biotoxicity risks 7. Second, the inherent brittleness and rigidity of most inorganic materials limit their application in flexible biomedical devices 8. More importantly, the limited charge output density and severe carrier recombination greatly restrict their therapeutic efficiency, while their single functionality has become a fundamental obstacle to meeting the demands of precision medicine 9, 10.

To overcome these limitations, the strategy of interface-engineered piezoelectric heterojunctions has emerged 11, 12. By combining a piezoelectric phase with materials like semiconductors, metals, or two-dimensional (2D) materials, heterojunctions can utilize the built-in electric field (BIEF) and unique band alignment at the interface to spatially guide charge flow. This configuration significantly promotes charge separation efficiency while suppressing recombination 13, 14. For example, Wang *et al.* 15 reported a BTO/MoS₂@CA core-shell structure whose catalytic performance under ultrasound was far superior to that of its single components. Furthermore, heterojunction design provides an ideal platform for multifunctional integration, enabling the combination of responses to multiple physical fields (light, sound, magnetism) with synergistic therapies, thus greatly enriching the therapeutic modalities and regulatory dimensions 16-18.

More revolutionarily, recent pioneering works have elevated the "interface" and "defects" themselves to a central role in the origin of the piezoelectric effect. Yang *et al.* discovered that in a metal-semiconductor Schottky junction, a device architecture that forms a rectifying barrier, a strong BIEF can break crystal symmetry. This induces a significant piezoelectric effect at the interface of a centrosymmetric semiconductor (like SrTiO₃) that is not inherently piezoelectric 19. Building on this, Park *et al.* 20 demonstrated the disruptive power of defect engineering: by rearranging oxygen vacancies in a centrosymmetric oxide, they created an equivalent piezoelectric response that was orders of magnitude stronger. These works expand the origin of the piezoelectric effect from the traditional "crystal structure" to the realm of "interface and defect engineering." This not only fundamentally validates the importance of the heterojunction strategy but also opens up entirely new avenues for the design of piezoelectric biomaterials.

**Figure 1:**
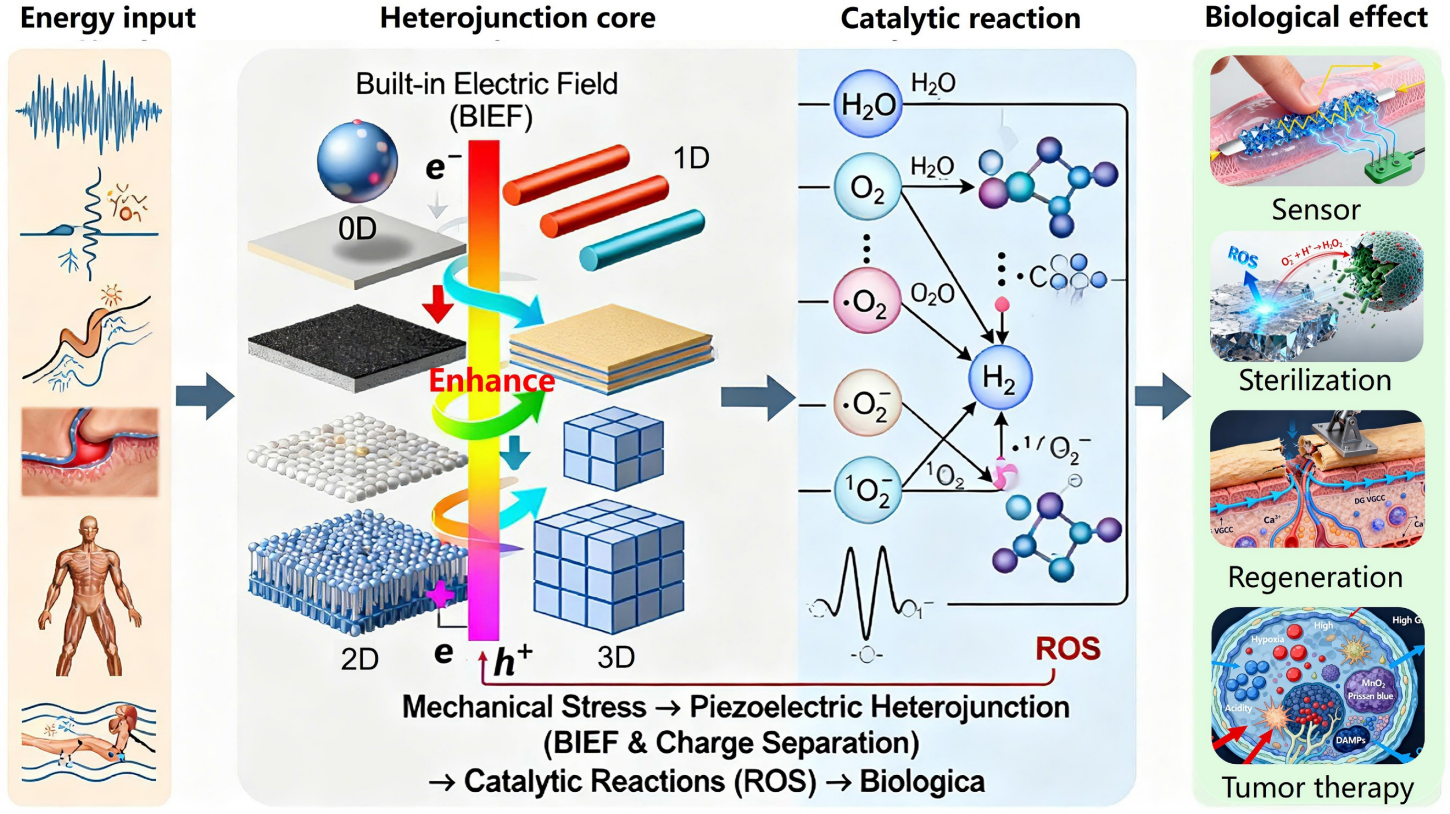
** Schematic diagram of the classification of piezoelectric heterojunctions and their application scenarios in the biomedical field;** applications in the biomedical field include efficient treatment of tumors, tissue repair and regenerative medicine, antibacterial and infection control, as well as sensors.

Given the rapid advancements in both the fundamental theory and applied research of piezoelectric heterojunctions, this review aims to systematically summarize their latest progress in the field of biomedical diagnostics and therapeutics. We will first elucidate their construction principles and enhancement mechanisms, focusing on the design concepts and performance characteristics of different types of heterojunctions. Subsequently, we will comprehensively review the cutting-edge applications of piezoelectric heterojunctions in biomedicine, including efficient cancer therapy, tissue repair and regeneration, antibacterial and infection control, and high-sensitivity biosensing. To ensure the comprehensiveness and timeliness of the content, we conducted a systematic search of major academic databases such as Web of Science, PubMed, and Scopus, using keywords including but not limited to 'piezoelectric heterojunction', 'piezocatalysis', 'sonodynamic therapy', 'tissue regeneration', and 'biosensor'. While our primary focus was on high-impact research published in the last five years to capture the latest advancements, we also performed a retrospective inclusion of foundational and landmark studies from earlier years to provide essential historical and mechanistic context. Finally, we will discuss the challenges and future directions of the field, aiming to provide a theoretical basis and design insights for creating a new generation of high-performance biomedical devices and to promote the clinical translation of this promising research area **(Figure 1)**.

## Construction principles and enhancement mechanisms of piezoelectric heterojunctions

The performance enhancement of piezoelectric heterojunctions hinges on their interface engineering, particularly the band alignment and the formation of the BIEF. These two mechanisms collectively determine charge separation efficiency, carrier lifetime, and multi-field coupling capability 21.

### Fundamentals of heterojunction interface engineering: Band alignment and BIEF

The efficacy of a piezoelectric heterojunction is fundamentally determined by its interfacial band alignment, which directly governs the separation, transport, and recombination behavior of photo-generated or piezo-generated charge carriers under external stimuli (e.g., mechanical stress, light illumination). Rational band engineering is key to achieving efficient charge separation, primarily classified into three classic types (Type-I, Type-II, Type-III) and some newly discovered special alignments (e.g., Type-V) 22, 23. The working principles, representative material systems, and their regulatory roles in charge separation for each band alignment type are detailed below.

#### Basic types of band alignment and charge separation mechanisms

Type-I (straddling gap) occurs when the conduction band (CB) and valence band (VB) of one material are completely encompassed within the bandgap of the other, forming a "nesting" structure. Both photo-generated electrons and holes migrate towards the material with the narrower bandgap, leading to spatial concentration of carriers in the same region. This is unfavorable for charge separation and promotes recombination. While unsuitable for catalysis, this structure allows rapid electron-hole recombination at the interface, making it applicable for light-emitting devices (e.g., LEDs) and lasers 24-26, but rarely used in PCM.

Type-II (staggered gap) refers to the staggered arrangement of the conduction and valence bands of the two materials at the interface. Typically, the CB and VB energy levels of one material are both lower than those of the other, forming a "staircase" structure. Photo-generated electrons spontaneously migrate from the higher CB to the lower CB, while holes migrate from the lower VB to the higher VB, achieving spatial separation of electrons and holes. This greatly suppresses recombination and provides more available carriers for catalytic reactions 27. In the n-TiO₂/BaTiO₃/p-TiO₂ sandwich heterojunction, a Type-II band structure is formed, achieving a charge separation efficiency as high as 86.6%, far exceeding that of pure TiO₂ (43.6%) 28. The 2D In₂Se₃/MoS₂ van der Waals (vdW) heterojunction also exhibits typical Type-II alignment. Due to the interlayer BIEF, its piezoelectric coefficient (d₃₃) is significantly enhanced 29.

Type-III (broken gap) occurs when the conduction band minimum of one material is lower than the valence band maximum of the other, resulting in completely misaligned bandgaps and band overlap at the interface. Electrons and holes can undergo tunneling, enabling separation, but interfacial recombination is usually rapid. This type is relatively uncommon in practical piezoelectric heterojunction design 30.

Recently, new band alignments have been discovered in some 2D material heterojunctions, such as Type-V alignment, found in partial van der Waals heterojunctions (e.g., PtSe₂/MoSe₂). Its valence band maximum (VBM) and conduction band minimum (CBM) are distributed within the same material, but unique delocalized states are formed through interlayer orbital coupling (e.g., Se-pz and Mo-dz² hybridization). This structure maintains high stability under external electric fields or strain, providing new ideas for designing highly stable piezoelectric optoelectronic devices 31.

#### Strategies for tuning band alignment

Band alignment is not immutable and can be precisely tuned through various means to optimize charge separation efficiency. For example, applying biaxial strain to MoS₂ can change its bandgap from direct to indirect and significantly alter the absolute values of the band edges, thereby affecting the heterojunction's band alignment type and charge separation efficiency 32. Applying an external vertical electric field can penetrate 2D materials and directly modulate their band structure, offering the possibility of dynamic, reversible tuning of band alignment 33. For 2D material heterojunctions, the band alignment exhibits significant angle and frequency dependence on the number of layers. For instance, in MoS₂ multilayer structures supported on glass substrates, both perfect absorption and strong coupling light-matter interaction mechanisms can be simultaneously achieved without complex nanostructures. MoS₂ flakes exceeding 10 layers can achieve perfect absorption of TM-polarized light, with the absorption condition determined by the interaction between excitons and Fabry-Pérot photon modes. In thick MoS₂ (e.g., 97 layers), B and C excitons strongly couple with photon modes, forming exciton-polaritons, and significant Rabi splitting is observed 34.

### Formation and enhancement mechanisms of the BIEF

The core physical basis for the performance improvement of piezoelectric heterojunctions lies in the formation and enhancement of the interfacial BIEF. This field is the key driving force for the efficient spatial separation of piezoelectric charges or photo-generated carriers and the suppression of recombination. Its strength and distribution directly determine the final output performance of the heterojunction. The formation of the BIEF primarily originates from charge rearrangement at the interface due to differences in the physical properties of the materials and can be significantly enhanced through various strategies 35, 36.

#### Origins of the BIEF

##### Contact potential difference and band bending

When two different materials (e.g., a piezoelectric material and a semiconductor) come into contact, their Fermi levels (E_F) must align at the interface to achieve thermodynamic equilibrium due to differences in their work functions and electron affinities. This process causes electrons to flow from the material with the smaller work function to the one with the larger work function until E_F is unified. Consequently, a space charge region (SCR) forms near the interface, accompanied by a corresponding built-in potential (V_bi) and band bending. The direction of this BIEF points from the semiconductor to the metal (for Schottky junctions) or from the n-type region to the p-type region (for p-n junctions), providing the core driving force for carrier separation 35, 37.

##### Contribution of piezoelectric polarization charges

The uniqueness of piezoelectric materials lies in their ability to generate significant polarization charges (direct piezoelectric effect) under applied mechanical stress 2. These bound charges appear on the material surface or interface and strongly couple with and modulate the original BIEF 38. For example, in a Schottky junction composed of a ZnO nanowire and an Au electrode, the positive polarization charges generated by applying compressive strain to ZnO can effectively lower the Schottky barrier height (SBH), significantly enhancing the electron injection efficiency from the semiconductor to the metal. This phenomenon, known as the piezo-electronic effect, was pioneered by Wang Zhonglin's team 39.

##### Interface polar symmetry breaking-induced piezoelectricity

Recent studies have found that even traditionally considered centrosymmetric semiconductors (e.g., SrTiO₃, TiO₂) can exhibit a piezoelectric response when forming a Schottky junction with a metal, due to atomic reconstruction and charge transfer at the interface leading to local symmetry breaking 38. For instance, the BIEF originating from interface band bending in heterostructures can induce polar symmetry, resulting in significant piezoelectric and pyroelectric effects 19.

Recently, new band alignments have been discovered in some 2D material heterojunctions, such as Type-V alignment, found in partial van der Waals heterojunctions (e.g., PtSe₂/MoSe₂). Its valence band maximum (VBM) and conduction band minimum (CBM) are distributed within the same material, but unique delocalized states are formed through interlayer orbital coupling (e.g., Se-pz and Mo-dz² hybridization). This structure maintains high stability under external electric fields or strain, providing new ideas for designing highly stable piezoelectric optoelectronic devices 31.

#### Strategies for tuning band alignment

Band alignment is not immutable and can be precisely tuned through various means to optimize charge separation efficiency. For example, applying biaxial strain to MoS₂ can change its bandgap from direct to indirect and significantly alter the absolute values of the band edges, thereby affecting the heterojunction's band alignment type and charge separation efficiency 32. Applying an external vertical electric field can penetrate 2D materials and directly modulate their band structure, offering the possibility of dynamic, reversible tuning of band alignment 33. For 2D material heterojunctions, the band alignment exhibits significant angle and frequency dependence on the number of layers. For instance, in MoS₂ multilayer structures supported on glass substrates, both perfect absorption and strong coupling light-matter interaction mechanisms can be simultaneously achieved without complex nanostructures. MoS₂ flakes exceeding 10 layers can achieve perfect absorption of TM-polarized light, with the absorption condition determined by the interaction between excitons and Fabry-Pérot photon modes. In thick MoS₂ (e.g., 97 layers), B and C excitons strongly couple with photon modes, forming exciton-polaritons, and significant Rabi splitting is observed 34.

### Formation and enhancement mechanisms of the BIEF

The core physical basis for the performance improvement of piezoelectric heterojunctions lies in the formation and enhancement of the interfacial BIEF. This field is the key driving force for the efficient spatial separation of piezoelectric charges or photo-generated carriers and the suppression of recombination. Its strength and distribution directly determine the final output performance of the heterojunction. The formation of the BIEF primarily originates from charge rearrangement at the interface due to differences in the physical properties of the materials and can be significantly enhanced through various strategies 35, 36.

#### Origins of the BIEF

##### Contact potential difference and band bending

When two different materials (e.g., a piezoelectric material and a semiconductor) come into contact, their Fermi levels (E_F) must align at the interface to achieve thermodynamic equilibrium due to differences in their work functions and electron affinities. This process causes electrons to flow from the material with the smaller work function to the one with the larger work function until E_F is unified. Consequently, a space charge region (SCR) forms near the interface, accompanied by a corresponding built-in potential (V_bi) and band bending. The direction of this BIEF points from the semiconductor to the metal (for Schottky junctions) or from the n-type region to the p-type region (for p-n junctions), providing the core driving force for carrier separation 35, 37.

##### Contribution of piezoelectric polarization charges

The uniqueness of piezoelectric materials lies in their ability to generate significant polarization charges (direct piezoelectric effect) under applied mechanical stress 2. These bound charges appear on the material surface or interface and strongly couple with and modulate the original BIEF 38. For example, in a Schottky junction composed of a ZnO nanowire and an Au electrode, the positive polarization charges generated by applying compressive strain to ZnO can effectively lower the Schottky barrier height (SBH), significantly enhancing the electron injection efficiency from the semiconductor to the metal. This phenomenon, known as the piezo-electronic effect, was pioneered by Wang Zhonglin's team 39.

##### Interface polar symmetry breaking-induced piezoelectricity

Recent studies have found that even traditionally considered centrosymmetric semiconductors (e.g., SrTiO₃, TiO₂) can exhibit a piezoelectric response when forming a Schottky junction with a metal, due to atomic reconstruction and charge transfer at the interface leading to local symmetry breaking 38. For instance, the BIEF originating from interface band bending in heterostructures can induce polar symmetry, resulting in significant piezoelectric and pyroelectric effects 19.

#### Strategies for enhancing the BIEF

##### Synergistic enhancement via multi-field coupling

Coupling the piezoelectric effect with other effects like the photogenerated effect or thermoelectric effect can enhance the BIEF multi-dimensionally.

Photo-Piezoelectric Coupling: In the n-TiO₂/BaTiO₃/p-TiO₂ sandwich heterojunction, the piezoelectricity of the BaTiO₃ layer is activated under ultrasonic vibration. The generated piezoelectric polarization field aligns with the direction of the original p-n junction's built-in field, synergistically increasing the charge separation efficiency from 43.6% for pure TiO₂ to 86.6%. Under 800 rpm stirring, the photocurrent density increased from 2.13 to 2.50 mA∙cm⁻² 28.

Applying compressive strain to a p-SnS/n-MoS₂ heterojunction not only modulated its Type-II band offset but also increased the depletion region width, thereby enhancing the BIEF. This ultimately resulted in a device photoresponse corresponding to a built-in potential of 0.95 eV 41.

##### Dimensionality and interface control

Two-dimensional materials, due to their atomically flat surfaces and lack of dangling bonds, can form high-quality van der Waals heterojunctions, greatly reducing carrier trapping by interface defects. For example, constructing core-shell structures (e.g., BaTiO₃/TiO₂) can effectively expand the interfacial contact area and allow the BIEF to distribute uniformly within the shell layer. Studies show that such structures can improve the piezocatalytic degradation efficiency of toluene several times compared to physical mixtures 42.

##### Defect and doping engineering

Artificially introduced defects (e.g., oxygen vacancies, OVs) or doping can effectively modulate the Fermi level and carrier concentration of materials, thereby altering the strength of the BIEF. In TiO₂-based heterojunctions, an appropriate amount of OVs can act as electron donors, increase n-type conductivity, make band bending steeper, narrow the space charge region, and increase the built-in field strength. The synergy between atomic Ni co-catalyst and OVs increased H₂ production by 4 times 43.

Beyond the classic heterojunction types, researchers have drawn inspiration from natural photosynthesis to design more complex Z-scheme heterojunctions, which can achieve stronger redox capabilities and higher charge separation efficiencies. For example, Ji's group employed a clever "edge modification" strategy to grow Bi₂O₃ in situ on the edges of 2D BiOCl nanosheets, constructing an in-plane BiOCl/Bi₂O₃ Z-scheme heterojunction 44. Under ultrasound, this system not only efficiently separates charges but also preserves holes with strong oxidizing power and electrons with strong reducing power. This allows it to catalyze a variety of reactions that are difficult for traditional catalysts to drive, effectively overcoming the limitations of the Tumor Microenvironment (TME). In another study, they used a similar strategy to construct a FeOCl/FeOOH Z-scheme heterojunction that achieved a "self-supplying H₂O₂" cascade catalysis. One component of the heterojunction oxidizes water to produce oxygen, while the other immediately reduces the generated oxygen to H₂O₂, providing a continuous supply of substrate for the subsequent Fenton reaction and greatly enhancing the therapeutic effect 45. These sophisticated designs based on Z-scheme heterojunctions represent a frontier direction for maximizing catalytic performance through the rational tuning of interfacial band structures and charge transfer pathways.

### Mechanistic controversies and a unifying perspective

Despite significant progress, the fundamental mechanism of piezocatalysis remains a subject of debate, primarily centered on two core issues.

One major point of contention is the origin of the active charges. Two competing models currently exist. The "band theory" model posits that the piezopotential modulates the semiconductor's band structure to separate internal charge carriers 35. In contrast, the "screening charge model" argues that the catalytic activity originates from the dynamic desorption of external screening charges adsorbed onto the polarized surface 35. These two models present a fundamental disagreement on the source of the charges. The "environmentally-mediated charge" model proposed by Xu *et al.*, which suggests a synergistic interplay between both mechanisms, offers a potential path toward unifying these two perspectives 46.

Furthermore, confounding effects introduced by the driving source, especially ultrasound, constitute another key controversy. The observed catalytic activity is often attributed entirely to piezocatalysis, but it may be the result of multiple coupled mechanisms. The ultrasonic cavitation effect, which is central to sonodynamic therapy (SDT), can independently drive sonocatalysis 18, 47. Concurrently, physical effects such as shockwaves or inter-particle friction could introduce competing tribocatalysis, or even negative synergistic effects 48. Therefore, accurately decoupling the respective contributions of piezoelectric, sonochemical, and triboelectric effects is a major experimental challenge.

In the face of these controversies, establishing a unifying framework is crucial. The piezocatalytic process can be viewed as a four-step sequence: (1) energy input, (2) multi-mode conversion, (3) charge separation, and (4) surface reaction. Within this framework, regardless of the initial energy conversion pathway, the core role of heterojunction engineering is to optimize the critical "charge separation" step. Future research must shift from simple "phenomenological attribution" to "mechanism-driven" design, systematically elucidating the contribution of each mechanism by combining experimental and theoretical methods.

## Classification of piezoelectric heterojunctions

Piezoelectric heterojunctions can be classified from multiple dimensions, including material dimensionality, composition/structure, interface bonding type, and functional properties. Current research frontiers primarily focus on low-dimensional piezoelectric heterojunctions, as they exhibit novel physical phenomena and superior performance not found in bulk materials.

### Classification by material dimensionality

#### 0D-2D piezoelectric heterojunction (Quantum dot/Nanodot compounded with 2D sheets)

**Figure 2:**
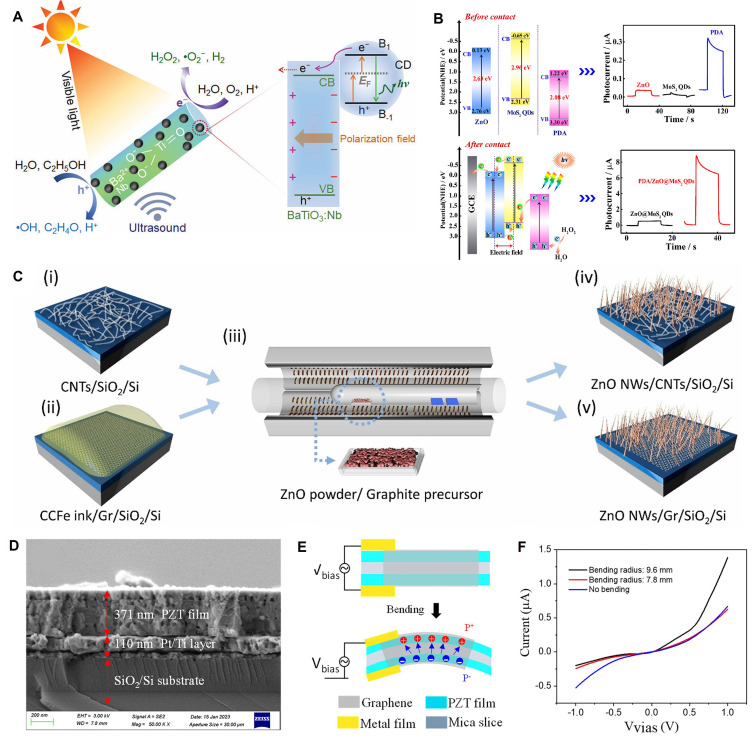
** Classification of piezoelectric heterojunctions. (A)** An illustration of the approach for catalytic production of H_2_O_2_ during simultaneous exposure to visible light and ultrasound with a catalyst based on BaTiO_3_:Nb and carbon quantum dots (CDs). Light irradiation of CDs results in the transfer of photoinduced electrons to the conduction band (CB) of BaTiO_3_:Nb, which in turn are involved in the reduction of O_2_. The electron donor C_2_H_5_OH quenches photoinduced holes (in the CDs and valance band [VB] of BaTiO_3_:Nb) and inhibits recombination of photoinduced charge carriers. Adapted with permission from 49, copyright 2022 The Authors. **(B)** PEC mechanism of ZnO, MoS_2_ QDs and PDA (before contact); the PEC mechanism of ZnO@MoS2 QDs and PDA/ZnO@MoS_2_ QDs (after contact). Adapted with permission from 50, copyright 2023 Elsevier B.V. **(C)** Heterostructure growth beginning with (i) a CNT sample and (ii) a graphene sample dip-coated in the CCFe ink followed by (iii) CVD synthesis of ZnO NWs using the double-tube method, resulting in (iv) ZnO NWs/ CNTs and (v) ZnO NWs/Gr heterostructures. Adapted with permission from 51, copyright 2021 by the authors. **(D)** SEM cross-section images of PZT film deposited on Si/SiO_2_/Pt/Ti and mica substrate. Adapted with permission from 59, copyright 2024 by the authors. **(E)** Working diagram of graphene/PZT device. Adapted with permission from 59, copyright 2024 by the authors. F) I-V output electrical transport test curve of 100 nm thickness. Adapted with permission from 59, copyright 2024 by the authors.

These materials involve uniformly loading zero-dimensional (0D) semiconductor quantum dots onto a 2D piezoelectric nanosheet substrate. The 0D piezoelectric nanodots can be carbon quantum dots (CDs), MoS₂ quantum dots, etc. For instance, Zhou *et al.* 49 sensitized Nb-doped tetragonal BaTiO₃ (BaTiO₃:Nb) with CDs. Piezoelectric polarization guides electrons to the semiconductor surface, limiting the recombination of photo-induced electron-hole pairs. The fastest generation of H₂O₂ was achieved with BaTiO₃:Nb/C (1360 μmol g_catalyst⁻¹ h⁻¹), which was 1.4 and 3.2 times faster than with BaTiO₃:Nb (972 μmol g_catalyst⁻¹ h⁻¹) and BaTiO₃/C (430 μmol g_catalyst⁻¹ h⁻¹), respectively **(Figure 2A)**. Xiao *et al.* 50 constructed a dual-sensitized PDA/ZnO@MoS₂ QDs biosensor. By coupling MoS₂ quantum dots to ZnO, the detection limit for miRNA-182-5p was as low as 0.17 fM, with good selectivity, stability, and reproducibility** (Figure 2B)**.

The advantages of such heterojunctions are that 0D nanodots provide a huge specific surface area, enabling dense interfacial contact with the 2D substrate. These interfaces are active sites for charge separation. The 2D sheet serves as an ideal supporting substrate, effectively preventing the agglomeration of 0D nanoparticles and fully exposing their active sites. Furthermore, 2D materials (especially graphene, MXene) often possess excellent conductivity, acting as "high-speed electron channels" to rapidly extract and transport charges generated by the 0D piezoelectric nanodots, greatly suppressing charge recombination. These heterojunctions are well-suited as efficient nano-sensitizers for tumor therapy or antibacterial applications, with the 2D sheet providing a good platform for biomolecule loading.

#### 1D-2D piezoelectric heterojunction (Nanowire compounded with 2D sheets)

These heterojunctions involve compounding one-dimensional (1D) piezoelectric nanowires (NWs) or nanorods (NRs) with 2D materials. The 1D material can grow or attach vertically or parallel to the 2D plane. Schaper *et al.* 51 used a direct two-step chemical vapor deposition (CVD) method to confirm the uniform growth of ZnO nanowires (ZnO NWs) on a single-walled carbon nanotube network and produced denser, vertically oriented ZnO NWs on a graphene film** (Figure 2C)**, suggesting potential applications in photocatalysts and biomedicine. In practical applications, the integration of PTO nanofibers with BOC nanosheets increased the contact area, amplified deformation, and enhanced the piezoelectric effect. Its photo-piezocatalytic efficiency was twice that of pure photocatalysis and three times that of piezocatalysis 52.

The characteristics of these materials are that 1D nanostructures possess excellent flexibility and mechanical strength, making them more prone to bending deformation under external force (e.g., ultrasound), thus generating a stronger piezoelectric potential. When combined with a 2D substrate, stress can be efficiently transferred throughout the heterojunction. Simultaneously, the 1D structure provides a longitudinal electron transport path, intertwining with the planar transport channels of the 2D material to form a three-dimensional, fast charge transport network, further promoting charge separation 53. These materials hold great potential in flexible or implantable self-powered sensing. The 1D-2D structure easily constructs porous, high-specific-surface-area flexible films that conform to human tissues and harvest tiny mechanical energy.

#### 2D-2D piezoelectric heterojunction (Van der Waals heterojunction)

These materials are formed by stacking two different two-dimensional materials through weak van der Waals forces and represent the current frontier and hotspot in 2D material research. For example, in a typical Type-II band alignment heterojunction (MoS₂/WS₂), photo-generated or piezo-generated electrons concentrate in the MoS₂ layer, while holes concentrate in the WS₂ layer, achieving intrinsic charge separation. Hole transfer from the MoS₂ layer to the WS₂ layer occurs within 50-100 fs after optical excitation 54, 55.

Traditional heterojunctions suffer from interface defects due to lattice mismatch, which trap charges. 2D-2D van der Waals heterojunctions are almost free from lattice mismatch constraints, forming atomically flat, dangling bond-free clean interfaces, greatly reducing charge recombination centers. By selecting different 2D materials (e.g., semiconducting MoS₂, metallic graphene, insulating h-BN) for combination 56, 57, the band alignment type (Type-I, II, III) of the heterojunction can be precisely designed to achieve directional charge separation. The electronic structure of such heterojunctions is highly sensitive to the number of layers, stacking angle, external electric field, or strain, offering the possibility for tunable, intelligent piezoelectric devices. Due to their ultra-thin, flexible, and transparent nature, 2D-2D heterojunctions have broad application prospects in wearable biosensors and micro/nano electromechanical systems (MEMS/NEMS), but large-scale preparation and transfer techniques remain challenging 58.

#### 3D-2D heterojunction (Bulk/Film compounded with 2D material)

These materials refer to the combination of three-dimensional (3D) piezoelectric bulk materials (e.g., ceramic plates, thick films) or thin films with 2D materials. The 2D material typically serves as a surface modification layer or an intermediate layer. For example, Bi *et al.* 59 integrated a PZT film onto a graphene substrate. With the generation of external force and strain gradient, the I-V output curve of graphene on the surface of the 100 nm thick PZT film exhibited rectifying characteristics due to the interfacial polarization mechanism** (Figure 2D-F)**.

These materials combine the advantages of strong piezoelectricity from 3D bulk materials and the unique surface properties (high conductivity, catalytic activity) of 2D materials. Modifying the surface of 3D piezoelectric ceramics with 2D materials (e.g., graphene) can improve surface chemistry, act as a charge interlayer to accelerate extraction, or serve as a protective layer to enhance biocompatibility and stability. They are mainly used for high-performance composite piezoelectric thick films or devices, such as high-precision ultrasonic transducers or implantable energy harvesting devices 60, 61.

#### 3D-3D heterojunction (Film compounded with film)

Heterojunctions formed by two three-dimensional materials (usually in thin film form). This is the most traditional form with the best compatibility with existing semiconductor processes. For instance, the carbon-silicon-carbon (C@Si@C) nanotube interlayer structure constructed by Liu *et al.* 62 had a capacity of ~2200 mAh g⁻¹ (~750 mAh cm⁻³), greatly exceeding commercial graphite anodes, and exhibited nearly constant Coulombic efficiency of ~98% over 60 cycles.

Such materials can be prepared using standard semiconductor processes like magnetron sputtering, pulsed laser deposition (PLD), and atomic layer deposition, facilitating large-area, uniform film integration. High-quality epitaxial or non-epitaxial interfaces can be obtained by precisely controlling deposition parameters. They are widely used in silicon-based MEMS biosensors for high-sensitivity detection of biomolecules **(Table 1)**.

### Classification by material composition and properties

#### Piezoelectric semiconductor-semiconductor type

These materials consist of two semiconductor materials, at least one of which possesses piezoelectric properties. This is the most abundant and extensively researched system. Examples include the classic n-ZnO/p-Si system, where ZnO is both a wide-bandgap semiconductor and a piezoelectric material, forming a p-n junction with silicon. Compared to bare Si NW electrodes, the n-ZnO/p-Si branched nanowires displayed photocurrent several orders of magnitude higher, and the doping concentration of the p-Si NW core influenced the photoelectrochemical cathode or anode behavior 63. n-TiO₂/p-NiO is another common all-oxide p-n junction piezoelectric heterojunction. The wide-bandgap TiO₂ is primarily responsible for generating electron-hole pairs, while the p-type NiO acts as a hole collection layer. The band matching between the two promotes charge separation, exhibiting high activity in piezocatalytic degradation of organic matter 64.

These materials form p-n junctions or heterojunctions. Due to differences in work function and electron affinity, band bending occurs at the interface of the two semiconductors, forming a BIEF. This built-in field synergistically couples with the piezoelectric polarization field generated upon excitation of the piezoelectric material, greatly promoting the spatial separation of photo-generated or piezo-generated electron-hole pairs and suppressing their recombination. They offer high charge separation efficiency, and the band structure can be flexibly tuned through doping. However, issues like lattice mismatch and thermal mismatch may exist; interface state defects can become charge recombination centers.

#### Piezoelectric material-semiconductor type

Composed of a strongly piezoelectric material (usually an insulator or wide-bandgap semiconductor) compounded with a conventional semiconductor material. For example, in PZT/MoS₂, the ferroelectric PZT provides a strong piezoelectric polarization field that can effectively modulate the carrier concentration and transport behavior in the conductive channel of the 2D semiconductor MoS₂, enabling the fabrication of piezoelectrically modulated transistor devices 65.

In these materials, strong piezoelectric materials generate extremely high piezoelectric potential under stress. This potential acts as a "gate voltage" to effectively modulate the interface barrier of the adjacent semiconductor i.e., the piezo-electronic effect. These heterojunctions exhibit strong piezoelectric output signals and significant modulation capability over the semiconductor channel. However, compatibility is poorer; strong piezoelectrics (e.g., PZT) often contain lead, raising biocompatibility concerns. They are currently mainly used for high-sensitivity mechanical sensing, e.g., electronic skin simulating human touch, with sensitivity far exceeding traditional sensors 66.

#### Piezoelectric-piezoelectric type

These heterojunctions are composed of two different piezoelectric materials. Examples include polymer/ceramic composites, which combine the advantages of polymers and ceramics, making them ideal for preparing flexible piezoelectric devices. BaTiO₃ nanoparticles dispersed in a PVDF matrix not only enhance the overall piezoelectricity but also induce the formation of more piezoelectric active β-phase in PVDF 67. Furthermore, PVDF/BaTiO₃ composites regulated osteogenesis in electrical stimulation experiments on human mesenchymal stem cells (hMSCs) 68.

These materials enable the superposition and coupling of piezoelectric effects. By designing the polarization directions of the two materials, their generated piezoelectric potentials can mutually enhance each other. More importantly, different piezoelectric materials may respond differently to stress of different frequencies or modes, potentially broadening the frequency response range or enabling more complex strain-to-electrical signal conversion. These heterojunctions achieve synergistic enhancement of piezoelectric performance, combining flexibility with high performance. However, polarization matching and stress transfer between different materials are design difficulties. They are ideal materials for flexible wearable energy harvesters and biomechanical sensors in biomedicine, capable of conforming to human skin or being implanted to harvest motion energy and monitor physiological signals.

#### Piezoelectric-metal type

These heterojunctions involve contact between a piezoelectric material and a metal, forming a Schottky junction or Ohmic contact. For example, in a ZnO Nanowire/Au electrode, when the ZnO nanowire is bent under pressure, positive and negative polarization charges are generated on the tensile and compressive sides, respectively. This asymmetrically changes the Schottky barrier at the metal-semiconductor contact, enabling a sensitive electrical response to external force 69. In BaTiO₃ @ Au NPs, the local surface plasmon resonance (LSPR) effect of Au nanoparticles can synergize with the piezoelectric effect. Under photoacoustic (PA) synergy, hot electrons generated by LSPR can inject into the conduction band of the piezoelectric material, greatly enhanced catalytic reaction (e.g., antibacterial, tumor therapy) efficiency 70.

In these heterojunctions, the metal primarily serves as an electrode for collecting and extracting piezoelectric charges. In the case of forming a Schottky junction, the polarization charges generated by the piezoelectric effect directly modulate the SBH, significantly affecting current transport characteristics. This is another important manifestation of the piezo-electronic effect. These heterojunctions offer high charge collection efficiency and sensitive Schottky junction modulation. However, the metal-piezoelectric interface may experience fatigue or delamination under repeated stress. They are the fundamental building blocks for all piezoelectric biosensors in biomedicine and are also widely used in piezocatalytic therapy systems, such as SDT **(Table 2)**.

The classification of piezoelectric heterojunctions is a multi-angle, intersecting system. For example, a heterojunction composed of a PZT film and MoS₂ can be simultaneously described as: a 3D-2D heterojunction, a piezoelectric material-semiconductor type heterojunction, and a modulation type or energy harvesting type heterojunction. This multi-dimensional classification method helps us understand its design concepts, preparation methods, and application potential from different perspectives. Current research frontiers mainly focus on low-dimensional (especially 2D-2D van der Waals) piezoelectric heterojunctions, as they exhibit many novel physical phenomena and excellent properties not found in bulk materials.

### Classification by interface bonding type

#### Epitaxial heterojunction

These heterojunctions are formed when one material (the epitaxial layer) grows on another single-crystal substrate with a highly ordered crystallographic arrangement. The core feature is the presence of chemical bonding (covalent, ionic) at the interface, requiring the lattice constants and thermal expansion coefficients of the two materials to be as matched as possible to reduce interface misfit dislocation density. For example, GaN/AlN is a classic epitaxial system; both have wurtzite structures with small lattice mismatch (~2.4%) and are widely used in high-performance optoelectronic devices 71.

Through lattice matching, atoms in the epitaxial layer are forced to "mimic" the lattice arrangement of the substrate, achieving atomically smooth connections. Common preparation methods include molecular beam epitaxy (MBE), PLD, and metal-organic CVD. The interface has scantly defects (dangling bonds, vacancies), greatly reducing non-radiative recombination centers for carriers, allowing efficient charge transport across the interface. However, material selection is limited as lattice-matched partners must be found; preparation costs are extremely high, requiring single-crystal substrates and complex vacuum equipment.

#### Van der waals heterojunction (vdW heterojunction)

These heterojunctions are formed by stacking two-dimensional materials (e.g., graphene, MoS₂, WSe₂) through weak van der Waals forces. This is a "non-epitaxial" integration method where no chemical bonds form at the interface, thus completely free from lattice mismatch constraints. Examples include MoS₂/WS₂ 54, 55, In₂Se₃/MoS₂ 29, etc. MoS₂/WS₂ is a typical Type-II band alignment where electrons and holes automatically separate into different layers.

These heterojunctions are created using mechanical exfoliation and dry/wet transfer techniques, stacking different 2D materials like "building blocks" in a designed sequence. CVD can also be used to directly grow multilayer heterostructures. Since the layers are bound by strong covalent bonds internally and weak van der Waals forces interlayer, the exfoliated surfaces have no dangling bonds, resulting in nearly perfect interfaces that significantly reduce charge scattering and recombination. They allow free combination of any 2D material regardless of lattice constant and symmetry differences (e.g., graphene/h-BN/MoS₂) 72, providing unprecedented freedom for band engineering and functional design. These materials hold great potential for biomedical applications. Their ultra-thin, flexible, and transparent nature makes them ideal candidates for next-generation wearable health monitoring sensors and low-dimensional implantable neural interfaces.

#### Mixed-dimensional heterojunction

This type of heterojunction is a broad classification referring to the non-epitaxial combination of materials with different dimensionalities (0D, 1D, 2D, 3D). The interfacial bonding force can be van der Waals forces, physical adsorption, or weak chemical bonds (such as coordination bonds). Examples include 0D-2D (e.g., MoS₂ quantum dots coupled to ZnO 50) and 1D-2D (e.g., ZnO nanowires grown on a graphene film 51).

These heterojunctions are fabricated by integrating nanomaterials of different dimensions using relatively low-cost methods such as solution-based techniques (e.g., spin-coating, drop-casting), electrophoretic deposition, and in-situ growth (e.g., hydrothermal methods), thus eliminating the dependence on single-crystal substrates and complex vacuum equipment. While they can combine the advantages of materials from each dimension, the interface quality is often uncontrollable, typically containing numerous defects and dangling bonds that can act as charge recombination centers. Furthermore, poor interfacial contact may affect stress transfer and charge transport. They have very broad applications in the biomedical field. Their solution-processability is highly suitable for preparing flexible biosensing membranes, porous tissue engineering scaffolds (with integrated piezoelectric stimulation), and injectable piezocatalytic nanomedicines (0D-2D systems).

The classification of piezoelectric heterojunctions is a multi-angled, intersecting system. From the perspective of interface bonding, epitaxial, van der Waals, and mixed-dimensional heterojunctions each have their own preparation characteristics and application advantages. The current research frontier is mainly focused on low-dimensional (especially 2D-2D van der Waals) piezoelectric heterojunctions, as they exhibit many novel physical phenomena and excellent properties not found in bulk materials.

In summary, by classifying piezoelectric heterojunctions across multiple dimensions, we can more systematically understand the relationship between their structure and performance. To directly link these structural advantages with catalytic performance, we have systematically summarized a series of representative studies from recent years and compiled their key performance parameters in Table 3. This table provides a detailed comparison of the structural types, piezoelectric responses, stimulation conditions, ROS generation capabilities, and final biological effects of different heterojunction systems.

A deep analysis of Table 3 provides strong data-driven support for the superiority of piezoelectric heterojunctions. For instance, in terms of piezoelectric performance, the CFO@BFO magnetoelectric heterojunction constructed by Ge *et al.* 79. utilizes the magnetostrictive effect to synergistically enhance the piezoelectric response, achieving an equivalent piezoelectric coefficient as high as 203 pm/V, far exceeding that of traditional single-component piezoelectric materials. In terms of catalytic efficiency, the construction of an Au@BaTiO₃ Schottky junction increased the ·OH production rate by 2.66 times compared to pure BaTiO₃ nanoparticles, a direct benefit of the enhanced charge separation promoted by the interfacial Schottky barrier. On the biological application level, the MoS₂/Bi₂MoO₆ heterojunction designed by Wang *et al.* 77. achieved an antibacterial rate of over 99% against Staphylococcus aureus under ultrasound stimulation, demonstrating its great potential as a highly efficient antibacterial agent.

This quantitative data not only provides conclusive evidence for the performance advantages of piezoelectric heterojunctions but also reveals the feasibility of tuning catalytic activity through interface engineering. It offers a crucial reference for the rational design of high-performance piezoelectric biomaterials tailored for specific diagnostic and therapeutic needs.

## Frontiers in biomedical applications of piezoelectric heterojunctions

Having systematically elucidated the construction principles, enhancement mechanisms, and multidimensional classification of piezoelectric heterojunctions, we can now appreciate the physicochemical basis for their enhanced performance. This chapter will focus on the cutting-edge applications of these remarkable materials in several biomedical fields, with a primary emphasis on their most representative application: efficient cancer therapy. We will systematically review how piezoelectric heterojunctions, through core mechanisms like piezocatalysis and in combination with various synergistic strategies, can achieve precise and effective diagnosis and treatment of diseases.

The roles that piezoelectric heterojunctions play in different biomedical applications are diverse, and consequently, the focus of material design varies significantly. To visually present this correspondence, Table 4 systematically summarizes the core challenges faced in mainstream application scenarios (such as cancer therapy, tissue regeneration, etc.) and lists the corresponding material design strategies and key performance parameters. This summary is intended to provide researchers with a clear reference framework to facilitate the efficient development of materials tailored for specific clinical needs.

### Efficient cancer therapy

As one of the leading causes of death worldwide, cancer and its treatment remain a central focus of medical research. Piezocatalytic therapy, an emerging paradigm that utilizes mechanical stimuli such as ultrasound to drive piezoelectric heterojunctions to kill tumor cells, has shown tremendous application potential. This approach has become a particularly noteworthy independent research direction for the treatment of deep-seated tumors, such as the notoriously difficult-to-treat glioblastoma (GBM). Several reviews have systematically reported on its progress and challenges 85.

#### Ultrasound-mediated piezocatalytic therapy

##### ROS-based synergistic catalysis

The core killing mechanism of ultrasound-mediated piezocatalytic therapy is the generation of ROS through the catalysis of water and oxygen molecules, which induces oxidative damage in tumor cells. The early strategy of using piezoelectric materials as sonosensitizers is referred to as SPDT 18. In this process, ultrasound excites the piezoelectric material to generate electron-hole pairs, and the heterojunction structure promotes their separation, thereby enhancing the efficacy of SDT 86. Current research has shifted its focus toward constructing multifunctional piezoelectric heterojunctions to maximize therapeutic effects by synergizing multiple catalytic mechanisms.

**Figure 3:**
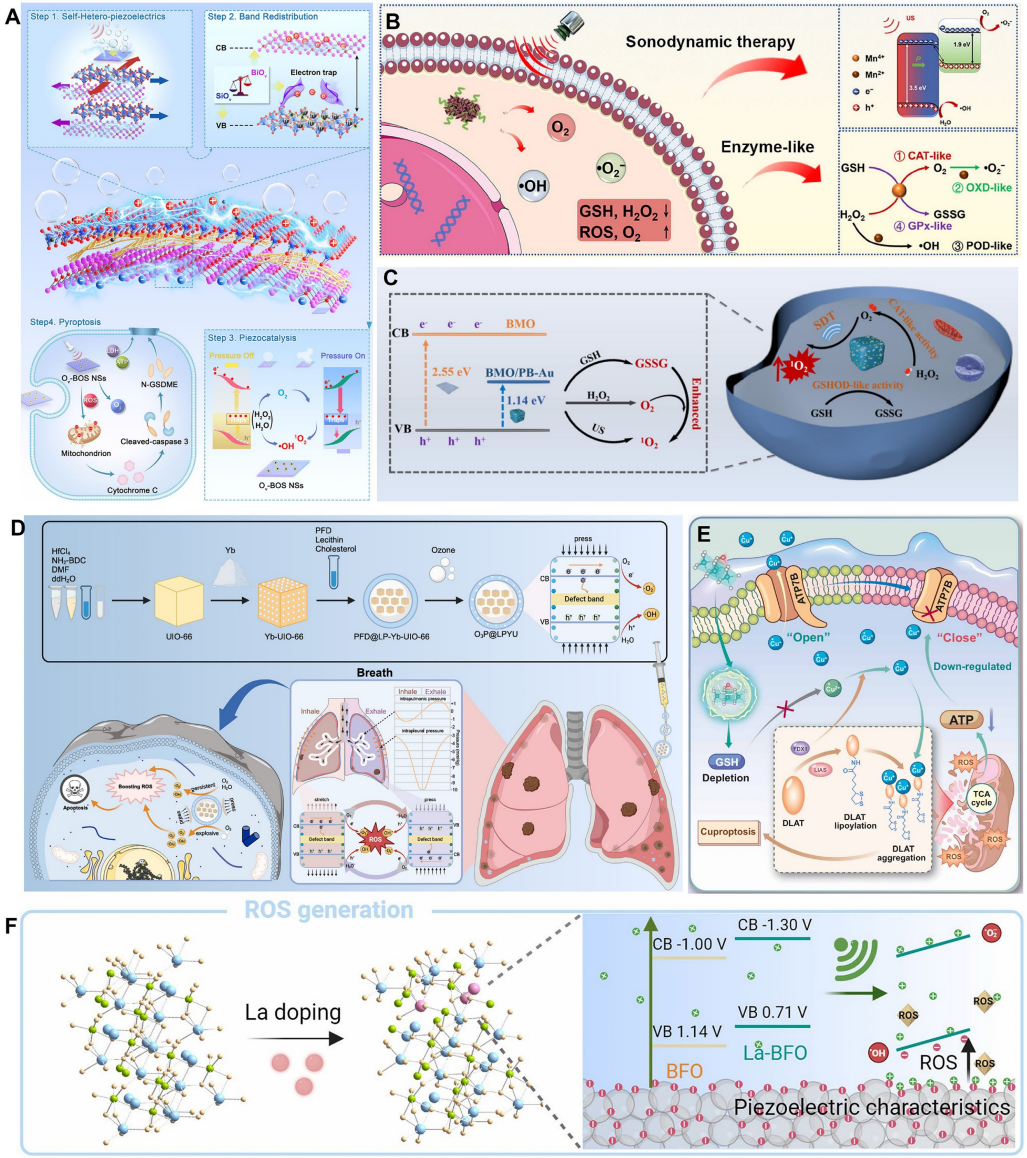
** Piezo-catalytic therapy. (A)** MechanismofOv-BOSNSs-mediated induction of tumor cell pyroptosis, consisting of four key steps. Step 1. The extraordinary piezoelectric performance caused by self-heterojunction accelerates and enhances charge carrier generation. Step 2. The band redistribution of Ov-BOS NSs caused by specific electron traps facilitates the spatial guided transport and stratified storage of sono-excited electrons and holes across the alternating [Bi_2_O_2_] and [SiO_3_] layers, respectively, thereby suppressing the recombination of charge carriers. Step 3. Synchronized sono-piezocatalytic pathways of Ov-BOS NSs boost the ROS generation. Step 4. Ov-BOS NSs impair mitochondrial function in cells, leading to caspase-3/GSDME-dependent pyroptosis. Adapted with permission from 73, copyright 2025 Wiley-VCH GmbH. **(B)** Schematic diagram of the piezoelectric M-BOC@SPNSs for enhanced SDT against cancer. Adapted with permission from 76, copyright 2023 Elsevier Inc. **(C)** The piezoelectric semiconductor acoustic sonosensitiser BMO/PB-Au@CM NPs that integrate superior CAT-like enzyme and special GSHOD-like enzyme into one system to achieve efficient antitumour SDT efficacy. Adapted with permission from 78, copyright 2024 Elsevier Inc. **(D)** Synthesis of O3P@LPYUNPs for enhancing physiological IPP-responsive piezoelectric catalytic therapy. NPs nanoparticles, IPP intrapleural pressure, NH2-BDC 2-amino-1,4-benzenedicarboxylic acid, DMF dimethylformamide, PFD perfluorodecalin, VB valence band, CB conduction band, ROS reactive oxygen species. Adapted with permission from 88, copyright 2025 The Author(s). **(E)** Schematic representation demonstrating the Fe- MoS2 enabled piezocatalytic and enzyocatalytic therapy synergistically induce tumor cell death through cuproptosis. Adapted with permission from 12, copyright 2025 The Authors. (F) La-doped BFO piezoelectric nanozyme triggers the enlarged ROS generation by lowering the band gap. Adapted with permission from 74, copyright 2025 The Author(s).

One major synergistic strategy is to integrate piezocatalysis with multiple enzyme-like activities. For example, Li *et al.* constructed an oxygen-vacancy-engineered bismuth silicate nanosheet (Ov-BOS NSs) with an alternating heterolayer structure 73. By introducing OVs, the material's piezoelectric strain coefficient (d₃₃) reached as high as 203 pm/V, significantly enhancing charge separation and ROS generation efficiency under ultrasound. Concurrently, the Ov-BOS NSs also possessed peroxidase- and catalase-like activities, enabling synergistic ROS production within the TME. This induced tumor cell pyroptosis via the caspase-3/GSDME pathway, achieving a tumor inhibition rate of 96.1%** (Figure 3A)**. Similarly, Zhao *et al.* constructed a manganese oxide (MnOx)-modified bismuth oxychloride (BiOCl) piezoelectric heterojunction 76. In this system, the MnOx component, through its multiple enzyme-like activities, synergistically depleted the intracellular antioxidant glutathione (GSH) and catalyzed the decomposition of hydrogen peroxide, thereby amplifying the oxidative stress generated by piezocatalysis. The system demonstrated good biocompatibility and a significant tumor suppression effect in a 4T1 breast cancer mouse model, with an inhibition rate of 70%** (Figure 3B)**.

Overcoming the barriers of the TME, particularly hypoxia and antioxidant defenses, is another critical synergistic strategy. Yang *et al.* constructed a multifunctional Bi₂MoO₆/Prussian blue-Au (BMO/PB-Au) piezoelectric nanoplatform that synergistically enhanced SDT efficacy through three mechanisms 78. First, the BMO/PB-Au heterojunction effectively promoted charge separation, increasing ROS yield. Second, the PB-Au component possessed both catalase (CAT)-like and glutathione oxidase (GSHOD)-like activities, which not only decomposed endogenous H₂O₂ to relieve hypoxia but also depleted GSH to weaken the tumor's antioxidant defenses. Finally, tumor targeting was achieved by cloaking the nanoparticles with cancer cell membranes (CM). This platform systematically addressed the traditional limitations of SDT by integrating piezocatalysis with chemical catalysis **(Figure 3C)**. Building on this, Zhang *et al.* combined this strategy with starvation therapy and nanomotors, constructing a Janus-structured hollow barium titanate-Au nanoparticle (C-hBT@Au-G) 87. In this design, the piezoelectric effect, a cascade enzyme catalysis (GOx/CAT), and nanomotor motion formed a self-enhancing cycle: the piezoelectric effect enhanced the enzyme catalytic efficiency, and the enzymatic product (O₂) in turn enhanced the piezodynamic therapy (PZDT) effect and propelled the nanomotor. This design broke through multiple drug delivery barriers, achieving highly efficient synergy between piezodynamic and starvation therapies.

In addition to the commonly used external ultrasound drive, utilizing endogenous physiological mechanical stress to activate piezocatalysis is an emerging frontier. Xu *et al.* innovatively developed a piezocatalytic nanozyme (O₃P@LPYU) activated by respiratory rhythm for the treatment of malignant pleural effusion (MPE) 88. By doping a metal-organic framework (MOF), UIO-66, with the rare-earth element ytterbium (Yb), they increased the material's d₃₃ from 67 pm/V to 256 pm/V. This system was able to use the pressure changes in the pleural cavity during the respiratory cycle (-3 to -10 mmHg) as a driving force to efficiently catalyze the generation of a large amount of ROS, inducing immunogenic death of tumor cells. In an MPE mouse model, the system showed excellent retention (half-life of 5 days) and therapeutic efficacy, with negligible systemic toxicity **(Figure 3D)**. Although there is considerable research on using self-powering or autonomous motion for energy harvesting 89, 90, sensing 91, and tissue repair 92, 93, reports of its direct application in cancer therapy are still scarce, representing a direction with great future potential.

##### Inducing ferroptosis and cuproptosis

Beyond the non-specific oxidative damage caused by ROS, a promising new strategy that has garnered significant attention in recent years is the use of piezoelectric heterojunctions to precisely induce specific forms of programmed cell death, such as ferroptosis, cuproptosis, and pyroptosis.

Zhang *et al.* 12 constructed a 2D molybdenum disulfide (MoS₂) piezocatalyst doped with single iron atoms (Fe-MoS₂). The single-atom iron doping effectively modulated the electronic structure and piezoelectric polarization behavior of MoS₂, significantly increasing its piezoelectric coefficient (d₃₃ enhanced from 6.16 pm/V to 9.22 pm/V). Under ultrasound, this material exhibited excellent piezocatalytic performance and also possessed multiple enzyme-mimicking activities, including peroxidase-, GSHOD-, oxidase-, and CAT-like activities. This combination synergistically produced a large amount of ROS while depleting GSH. The ROS burst and GSH depletion collectively disrupted intracellular copper ion homeostasis, inhibited ATP7B function, and promoted the accumulation of endogenous copper ions in mitochondria. This, in turn, induced cuproptosis, a form of copper-dependent cell death, while synergistically triggering ferroptosis and ferritinophagy. The material demonstrated significant tumor suppression effects in both *in vitro* and *in vivo* experiments. This study represents the first strategy for piezocatalytically inducing cuproptosis without an external copper source, providing a new paradigm for the application of piezoelectric materials in cancer therapy and broadening the design of cuproptosis-related nanomedicines** (Figure 3E)**. In a different approach, Zhong *et al.* 75 used a 2D copper-based substrate for a MOF. Through coordination with dimethylimidazole, the resulting CM was endowed with piezoelectric properties. Under ultrasound, the copper ions in the CM could induce cuproptosis in tumor cells, which, combined with the generated ROS, accelerated cell death.

Li *et al.* 74 innovatively designed a lanthanum (La)-doped bismuth ferrite (La-BFO) piezoelectric nanozyme. Through a synergistic strategy of specific element doping and vacancy engineering, they achieved efficient induction of pyroptosis in breast cancer cells. The core features of this system were twofold: the introduction of La not only narrowed the bandgap of BFO (from 2.14 eV to 2.01 eV) and created OVs, significantly enhancing the efficiency of ROS generation (including ·OH, ·O₂⁻, and ¹O₂) under ultrasonic excitation; concurrently, the released La³⁺ ions could specifically damage the lysosomal membrane, synergizing with ROS to activate the Caspase-1/GSDMD pathway. This dual amplification of the pyroptotic effect overcame the apoptosis resistance of tumor cells. Both *in vitro* and *in vivo* experiments confirmed that the system effectively inhibited tumor growth (the intratumoral injection + ultrasound group achieved an inhibition rate of 76.7%) and enabled computed tomography (CT)/magnetic resonance imaging (MRI) dual-modal imaging guidance, thanks to the presence of Bi and Fe elements. This work provides a new paradigm for tuning the performance of piezoelectric materials through elemental doping and for combining ion therapy with catalytic therapy to induce inflammatory cell death **(Figure 3F)**.

#### Piezocatalytic immunotherapy

Piezocatalytic immunotherapy 17, 94, which combines piezocatalysis with immunotherapy, is an emerging and highly promising direction in recent years. Its core strategy is to convert immunologically suppressive "cold tumors" into immunologically active "hot tumors" through a dual-action mechanism. On one hand, piezocatalysis generates ROS to induce ICD, releasing tumor antigens to initiate an immune response. On the other hand, the local micro-electric field or related biological effects generated by the piezoelectric effect can directly modulate the TIME. This section will review the latest progress from these two aspects.

##### Inducing immunogenic cell death

Inducing ICD in tumor cells is the crucial first step in activating anti-tumor immunity. In addition to traditional apoptosis, growing evidence suggests that specific forms of programmed cell death, such as ferroptosis, pyroptosis, and PANoptosis, are also highly immunogenic. For example, research by Cheng *et al.* 95 demonstrated that ferroptosis induced by a Bi₂MoO₆-MXene piezoelectric heterojunction could effectively promote the release of damage-associated molecular patterns, thereby activating downstream dendritic cell maturation and anti-tumor immunity. Therefore, precisely regulating cell death pathways via piezocatalysis is a core strategy for achieving efficient piezocatalytic immunotherapy.

**Figure 4:**
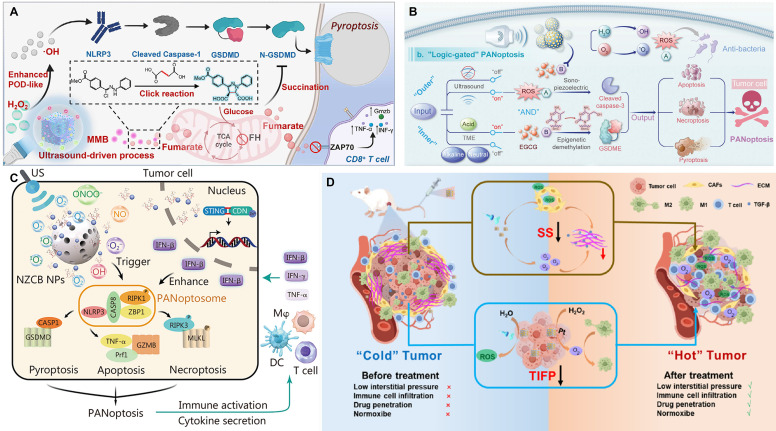
** Piezo-catalytic immunotherapy. (A)** Schematic illustration of US-driven BFTM-induced ROS triggering pyroptosis and amplification of pyroptosis degree through bioorthogonal reaction consumption of fumarate. Adapted with permission from 96, copyright 2025 Wiley. **(B)** The concrete mechanisms of “outer sono-piezoelectric and inner demethylation regulation” PANoptosis activation. Adapted with permission from 100, copyright 2024 Wiley. **(C)** Schematic illustration of tumor PANoptosis pathway and immune activation. Adapted with permission from 101, copyright* 2024 Wiley-VCH GmbH*. **(D)** A schematic diagram illustrates how piezoelectric catalytic water splitting reduces TIFP and induces apoptosis in CAFs, leading to a reduction in SS and enhanced immune cell infiltration. Adapted with permission from 106, copyright 2025 Published by Elsevier Inc.

Targeting pyroptosis, Niu *et al.* 96 constructed an ultrasound-driven, piezo-enhanced iron-based single-atom nanozymeco-loaded with triphenylphosphine and the bioorthogonal fumarate reagent MMB. Under ultrasound, the piezoelectric effect of BFTM significantly enhanced the peroxidase-like activity of the single iron sites, generating a large amount of ·OH to induce caspase-1/GSDMD-mediated pyroptosis. Simultaneously, MMB consumed intracellular fumarate via a 1,3-dipolar cycloaddition reaction. This not only prevented the succination of the key cysteine in GSDMD to enhance pyroptosis but also restored ZAP70 phosphorylation to normalize the T-cell receptor signaling pathway, thereby revitalizing CD8⁺ T-cell function **(Figure 4A)**. Cai *et al.* 97 constructed a biodegradable manganese-doped hydroxyapatite (Mn-HAP). Under ultrasonic excitation, the released Ca²⁺ and ROS promoted pyroptosis, while Mn²⁺ activated the cGAS-STING pathway, triggering innate immunity and further enhancing the pyroptosis-induced immunotherapeutic effect. Wu *et al.* 98 constructed a narrow-bandgap Ir-C₃N₅ nanocomposite by coordinating Ir(tpy)Cl₃ onto nitrogen-rich C₃N₅ nanosheets, which significantly enhanced the material's piezocatalytic performance and oxygen self-supply capability. Under ultrasound, the composite efficiently generated ROS and catalyzed the decomposition of endogenous H₂O₂ into O₂, alleviating the hypoxic TME. The Ir-C₃N₅ targeted lysosomes, where the piezoelectric effect induced lysosomal rupture, inhibited autophagy, and activated the caspase-1/GSDMD-N-mediated pyroptosis pathway. This process released DAMPs, induced ICD, activated both innate and adaptive immune responses, significantly inhibited primary, distant, and metastatic tumor growth, and established long-term anti-tumor immune memory**.**

Pyroptosis, apoptosis, and necroptosis are collectively referred to as PANoptosis 99. Xu *et al.* 100 developed a multifunctional nanocatalyst based on mesoporous piezoelectric barium titanate (mtBTO), named NZCB NPs. Under ultrasound excitation, it could efficiently generate reactive oxygen and nitrogen species (ROS/RNS), such as ·O₂⁻, NO, and ONOO⁻, thereby inducing highly immunogenic PANoptosis in B16 melanoma cells. The nanosystem could also release the STING agonist CDN and Zn²⁺, synergistically activating innate and adaptive immune responses, promoting M1-type macrophage polarization and DC maturation, and remodeling the TME **(Figure 4B)**. Meanwhile, Wang *et al.* 101 constructed a composite scaffold (STE-BG) based on an "external piezoelectric-internal epigenetic" logic gate. By combining porous piezoelectric SrTiO₃ nanoparticles (MeST) loaded with the epigenetic modulator EGCG with a 3D-printed bioactive glass scaffold, they achieved precise PANoptosis induction and immunotherapy for osteosarcoma, with a tumor inhibition rate of approximately 73.47% **(Figure 4C)**. Zhong *et al.* 102 developed a manganese-doped hafnium oxide (HMO, 20% Mn) piezoelectric nanocatalyst. The Mn doping introduced OVs, reduced the bandgap, and enhanced the piezoelectric response. In the TME, it synergistically amplified oxidative stress through dual piezocatalytic and enzymatic mechanisms, depleting GSH and alleviating hypoxia to induce PANoptosis. This process was accompanied by the release of ICD markers calreticulin (CRT) and high-mobility group box 1 (HMGB1), which significantly promoted DC maturation, CD8⁺ T-cell infiltration, and pro-inflammatory cytokine secretion, ultimately achieving significant tumor suppression and activating a systemic anti-tumor immune response both *in vivo* and *in vitro*. These studies all utilize US to activate nanomaterials to generate ROS, induce immunogenic cell death (ICD) (PANoptosis or pyroptosis), and activate anti-tumor immunity. They showcase the synergistic therapeutic effect of piezocatalysis in achieving PANoptosis, providing a new paradigm for piezoelectric nanocatalytic immunotherapy.

##### Remodeling the tumor immune microenvironment

High tumor interstitial fluid pressure (TIFP) obstructs the infiltration of immune cells. Immune cells cannot effectively move "against" this high pressure to enter the tumor core, leading to the formation of an "immune desert" or an "immune-excluded" microenvironment. Even when T-cells in the peripheral blood are activated by immune checkpoint inhibitors (ICIs), such as PD-1/PD-L1 antibodies, these activated cells cannot enter the "battlefield," resulting in immunotherapy failure 103-105. Zhang *et al.* 106 constructed an AgNbO₃/Pt@HA (ANPH) piezoelectric heterojunction system. Under US, the piezocatalytic reaction decomposed water molecules in the tumor interstitial fluid to produce H₂ and ·OH, reducing the TIFP by 48.16%. Simultaneously, the generated ROS induced apoptosis in cancer-associated fibroblasts (CAFs) (up to 59.4%), reducing the extracellular matrix (ECM) and solid stress by 44.07%. This significantly promoted the infiltration of CD8⁺ and CD4⁺ T-cells (by 3.95-fold and 3-fold, respectively), successfully converting a "cold tumor" into an immunogenic "hot tumor" **(Figure 4D)**. The mechanism also included the Pt nanozyme-catalyzed decomposition of H₂O₂ to produce oxygen, the promotion of M2-type macrophage polarization to the M1 type, and the induction of ICD. Ultimately, this system suppressed the growth of primary, distant, and metastatic tumors, demonstrating good biocompatibility and systemic anti-tumor immune efficacy.

Secondly, reversing immunosuppression can be achieved by directly modulating the function of key immune cells or the metabolic state of the microenvironment. On one hand, studies have shown that the micro-electric field or related biological effects generated by piezoelectric materials under mechanical stimulation can effectively induce the polarization of pro-tumoral M2-type tumor-associated macrophages (TAMs) to the anti-tumoral M1 phenotype by activating specific signaling pathways (such as Ca²⁺-mediated pathways) 17. On the other hand, reprogramming tumor immune metabolism is a more advanced strategy. Lactate accumulation in the TME is a key metabolite causing immunosuppression. Recently, Fan *et al.* 107 developed an ingenious biohybrid microrobot that utilizes the innate lactate-consuming ability of Veillonella atypica and combines it with the piezocatalytic effect of BTO to accelerate this metabolic process. By efficiently clearing local lactate in the tumor, this system successfully repolarized M2 macrophages to the M1 phenotype, promoted DC maturation, and restored T-cell activity, providing a completely new approach for achieving piezo-immunotherapy through metabolic regulation.

To further explore innovative drug delivery routes and synergistic strategies, researchers have begun to combine piezocatalysis with bio-microrobotics. For example, Ji's group has continuously reported on biohybrid microrobots for the oral treatment of colorectal cancer in Advanced Materials and Science Advances 45,107. In their series of works, they loaded BaTiO₃ piezoelectric nanoparticles onto the surface of bacteria that can enrich in the hypoxic TME and encapsulated them in enteric microcapsules. After oral administration, the microrobots utilized the active targeting ability of the bacteria to enrich and achieve deep penetration at the intestinal tumor site. Under external US triggering, the piezocatalytic effect of BaTiO₃ not only produced reactive species to directly kill tumor cells but also synergized with bacterial metabolism to efficiently consume the immunosuppressive metabolite—lactate—in the TME. This "physical catalysis + biological metabolism" dual lactate-clearing strategy successfully reprogrammed pro-tumoral M2 macrophages into anti-tumoral M1 macrophages, promoted DC maturation, and ultimately activated a systemic T-cell immune response capable of inhibiting distant metastases. This series of pioneering works provides a new oral delivery paradigm for piezo-immunotherapy and demonstrates the great potential of synergizing with living microorganisms to remodel the TME.

Finally, immune cell activity can be restored by improving the chemical state of the microenvironment. Tumor hypoxia is one of the main factors inhibiting the function of cytotoxic T-cells. Many of the oxygen-producing piezoelectric heterojunctions discussed in Section 4.1.1.1, such as BMO/PB-Au 78 and Ir-C₃N₅ 98, also have mechanisms that include improving the immune microenvironment by alleviating hypoxia. This strategy can effectively restore the killing function of T-cells and produce a powerful synergistic effect with therapies such as ICIs.

Piezo-catalytic immunotherapy, by coupling US-excited piezoelectric effects with the body's immune system, marks a significant shift in the tumor treatment paradigm from mere physicochemical killing to immune activation. Although this strategy demonstrates a "two-pronged" synergistic advantage by generating ROS to induce ICD and utilizing local electric fields to directly regulate immune cell function, and has successfully observed the reversal of "cold tumors" to "hot tumors" in various models, its underlying mechanisms still have significant limitations. Current research predominantly focuses on phenomenological observations. There is a lack of detailed molecular interpretation at the molecular level regarding how specific electrical signal parameters (e.g., intensity, frequency, waveform) generated by piezoelectric materials are precisely "decoded" to specifically activate key immune signaling pathways (e.g., cGAS-STING, Ca²⁺-NFAT). Meanwhile, the universality, durability, and reproducibility of this immune activation effect across different tumor models remain to be systematically validated. How to serialize and personalize combine this efficient anti-tumor immune response with clinically existing strategies like immune checkpoint inhibitors, and overcome the obstacles of the immunosuppressive microenvironment, are the core challenges in moving from proof-of-concept to clinical translation.

#### Multimodal synergistic therapy (e.g., Sonodynamic-chemo/photothermal therapy)

Although piezocatalytic immunotherapy shows great therapeutic potential by activating the body's immune system, its single-mechanism approach still faces inherent bottlenecks such as tumor heterogeneity, an immunosuppressive microenvironment, and individual response variability. To overcome these limitations, research focus has shifted to multimodal synergistic therapy strategies. The organic combination of piezocatalysis with other modalities like sonodynamic, chemo-, and photothermal therapies aims to overcome the shortcomings of monotherapy through spatially and temporally synchronized, multiple killing mechanisms. This not only directly enhances the clearance efficiency of primary tumors but can also reverse the immunosuppressive state by modulating the TME and inducing a stronger ICD, thereby synergistically amplifying the anti-tumor immune response. We prefer to term such research as sensitization strategies for piezocatalytic therapy.

"Theranostics," which combines diagnostic imaging with precision therapy, is one of the ultimate goals of multimodal synergistic strategies. However, integrating modules with excellent imaging capabilities (like fluorescent probes) with piezoelectric therapeutic modules on the same nanoplatform is a major challenge, as most piezoelectric materials are opaque and can quench fluorescent signals. To address this, Ullah *et al.* 108 provided a solution through an ingenious core-shell structural design. They used down-conversion nanoparticles emitting in the second near-infrared (NIR-II) window as the "core" and coated them with a piezoelectric "shell" of potassium niobate (KNbO₃) modified by defect engineering (gadolinium ion doping). The cleverness of this design lies in the fact that gadolinium doping not only increased the piezoelectric coefficient tenfold and endowed the material with MRI contrast capabilities but also widened the bandgap of the piezoelectric shell, making it highly transparent in the NIR-II window. This allowed the fluorescence signal from the core to penetrate without obstruction. Ultimately, the platform, biomimetically camouflaged with a cell membrane, successfully achieved highly efficient PZDT for glioma guided by NIR-II fluorescence/MR dual-modal imaging. This work, published in Small, provides important design principles for developing high-performance piezoelectric theranostic nanoplatforms.

**Figure 5:**
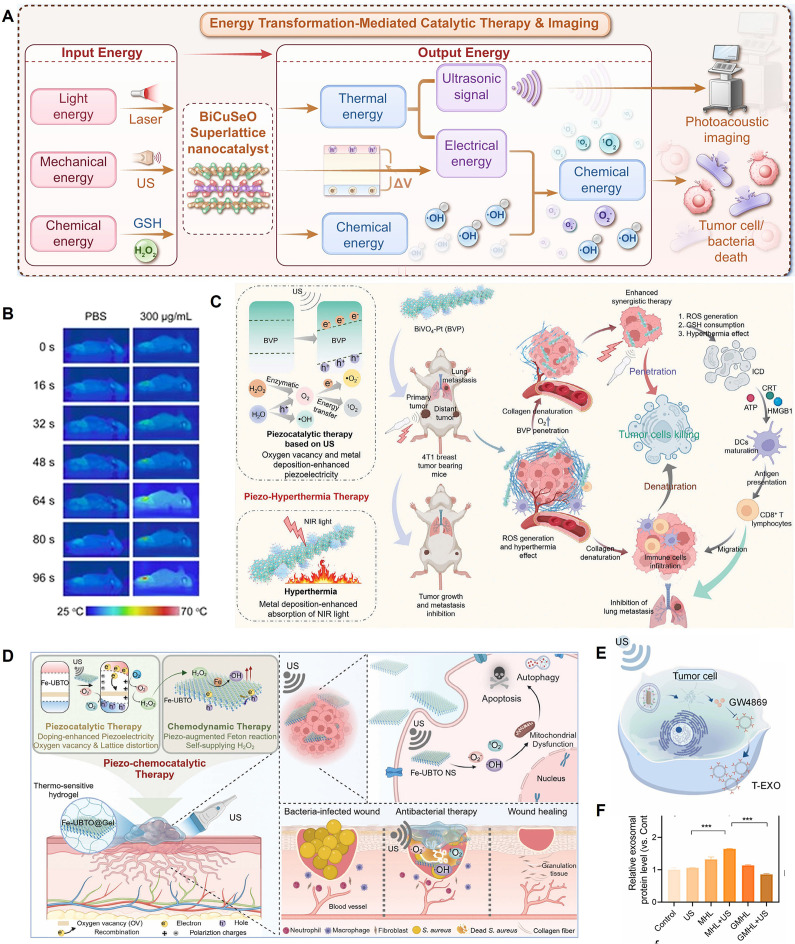
** Synergistic Therapy. (A)** Schematic illustration of the multifaceted energy conversion effects based on superlattice 2D BCSO nanocatalysts for theranostic applications. Adapted with permission from 80, copyright 2025 The Author(s). **(B)** photographic images after co-incubation with erythrocytes. Adapted with permission from 109, copyright 2023 Wiley. **(C)** Schematic diagram of the “denaturation” and “penetration” strategy based on BVP used to enhance tumor piezo-thermal-immune synergistic therapy. Adapted with permission from 110, copyright 2024 Wiley. **(D)** Schematic illustration of the fabrication of Fe-UBTO NSs and their synergistically piezo-chemocatalytic efficacy under US activation for antitu mor and antibacterial therapies. Adapted with permission from 113, copyright 2024 Wiley-VCH GmbH. **(E)** Scheme showing the inhibition of tumor-derived exosome secretion by GMHL under US stimulations. Adapted with permission from 114, copyright 2025 The Author(s). **(F)** Determination of exosomal protein content in cell supernatants after different treatments by BCA kits. Adapted with permission from 114, copyright 2025 The Author(s).

Zhang *et al.* 80 innovatively developed 2D BiCuSeO superlattice nanosheets (BCSO NSs) as a nan catalyst driven by multimodal energy conversion. Its core feature is the integration of photothermoelectric (PTE) catalysis, sono-piezoelectric catalysis, and enzyme-like catalysis in a single system for the first time, leveraging the material's natural layered heterostructure (alternating [Bi₂O₂]²⁺ and [Cu₂Se₂]²⁻ sublayers) and narrow bandgap (~1.04 eV). Theoretical calculations and COMSOL simulations showed that its internal electric field could significantly promote charge separation and migration under photothermal cycling or mechanical pressure. Experiments confirmed that BCSO NSs could efficiently generate •O₂⁻ and ¹O₂ through photothermal-electric-chemical energy conversion under NIR-II laser excitation, while also possessing PA imaging capabilities, demonstrating high antibacterial performance in an infected wound model. Under US activation, the piezoelectric effect synergized with peroxidase (POD)-like activity to not only catalyze the generation of •OH but also deplete GSH, disrupting the redox homeostasis in tumor cells and inducing apoptosis and ferroptosis. This significantly inhibited tumor growth and prolonged survival in both melanoma and orthotopic liver cancer models **(Figure 5A)**. Meanwhile, Yang *et al.* 109 designed piezoelectric BiO₂₋ₓ nanosheets (NSs) rich in OVs. Under US excitation, they initiated a cascade reaction to generate ROS, while the US-induced electron motion also triggered a sonothermal effect, rapidly increasing the temperature to 65 °C under low-power (1.2 W/cm^2^) and short-duration (96 s) US irradiation. This achieved a multimodal synergistic therapy of piezocatalysis, enzyme catalysis, and sonothermal therapy for tumors **(Figure 5B)**. Wang *et al.* 110 constructed bismuth vanadate nanorods decorated with platinum nanodots as an oxygen-deficient piezoelectric-photothermal sensitizer. Under synergistic activation by near-infrared (NIR) light and US, the Pt nanodots not only enhanced the photothermal conversion efficiency (PCE) (31.2%) but also catalyzed the decomposition of overexpressed H₂O₂ in the TME into O₂, alleviating tumor hypoxia and reversing the immunosuppressive state. This strategy significantly inhibited the growth of primary and distant tumors (inhibition rates of 72% and 75%, respectively) and lung metastasis. RNA sequencing confirmed its modulation of pathways such as P53, HIF-1, and the TCR, remodeling the immune microenvironment and providing a new approach for reversing TME immunosuppression through piezo-photothermal-immune synergistic therapy **(Figure 5C)**. Xu *et al.* 111 constructed a multifunctional nanoplatform based on tetragonal BaTiO₃, named BTAPs. By depositing ultrasmall Au nanoparticles (Au NPs) on its surface and coating it with polydopamine (PDA), they achieved a synergistic effect of enhanced piezocatalytic therapy and photothermal therapy (PTT) under US activation, along with CT imaging capability. The introduction of Au NPs significantly enhanced the piezoelectric performance of BT (piezoelectric constant increased from 15.5 to 37.5 pm/V), promoting the separation of electron-hole pairs under US and thus efficiently generating ROS (mainly •OH) to induce tumor cell apoptosis. The PDA coating not only improved the material's biocompatibility and stability but also endowed it with an excellent PCE (24.9%).

Chen *et al.* 112 constructed a piezocatalytic nanocomposite BTO-Pd-MnO₂-HA with TME-modulating functions to enhance the synergistic SDT and chemodynamic therapy (CDT) for melanoma. This material features BaTiO₃ nanocubes as the core, with deposited Pd nanoparticles forming a Schottky junction. The outer MnO₂ shell degrades in the acidic TME in response to H₂O₂ and GSH, releasing Mn²⁺ ions to catalyze a Fenton-like reaction that generates •OH, while simultaneously consuming GSH and producing O₂ to alleviate tumor hypoxia. The outermost hyaluronic acid layer provides active targeting to CD44-overexpressing melanoma cells, offering good biocompatibility and targeting. Jing *et al.* 113 constructed atomically thick, ultrathin Bi₄Ti₃O₁₂ nanosheets (Fe-UBTO NSs) that exhibited synergistic piezoelectric-chemodynamic catalytic activity under US activation for the treatment of skin melanoma and the healing of bacteria-infected wounds. The doped Fe ions have Fenton-like catalytic activity, utilizing the overexpressed H₂O₂ in the tumor and bacterial infection microenvironments to produce highly toxic hydroxyl radicals (•OH). The US-driven piezoelectric effect not only self-supplied H₂O₂, but also accelerated the Fe²⁺/Fe³⁺ cycle, further enhancing •OH generation, thereby effectively inhibiting tumor growth. It also possessed potent antibacterial capabilities and accelerated infected wound healing by promoting angiogenesis and collagen deposition** (Figure 5D)**.

Qu *et al.* 114 constructed a piezocatalytic nanoplatform by doping HAP with manganese ions (Mn²⁺) and loaded it with the exosome inhibitor GW4869. US activation of the piezoelectric effect generated ROS, which triggered the cleavage of a ROS-cleavable lipid (DSPE-TK-mPEG) to release GW4869. This inhibited the secretion of tumor-derived exosomes (especially PD-L1⁺ exosomes) induced by US, thereby alleviating immunosuppression **(Figure 5E-F)**. GMHL significantly inhibited tumor growth in multiple tumor models, promoted the maturation of DCs, the infiltration of cytotoxic T lymphocytes and natural killer cells, and increased the expression of immune factors like tumor necrosis factor-alpha (TNF-α) and interferon-gamma (IFN-γ), thus synergistically enhancing the anti-tumor immune response.

In addition to synergizing with other therapies, utilizing multi-physics coupling (such as magneto-electric coupling) is another important enhancement strategy. Ge *et al.* 79 constructed a core-shell magnetostrictive-piezoelectric nanocatalyst (CoFe₂O₄@BiFeO₃, CFO-BFO NPs). The magnetostrictive core (CFO) generated strain under an alternating magnetic field, which drove the polarization of the piezoelectric shell (BFO). This produced a local electric field on the nanoparticle surface, catalyzing water and oxygen to generate ROS such as •OH and superoxide anions (•O₂⁻). This material achieved magnetically controlled, deep-tissue-penetrating, and non-invasive ROS generation without the need for external chemical sacrificial agents or light/sound stimulation, significantly inducing tumor cell apoptosis and effectively inhibiting tumor growth *in vivo*. They validated the magneto-electro-chemical coupling mechanism and its catalytic activity using finite element modeling, piezoelectric force microscopy, and electron paramagnetic resonance, demonstrating excellent anti-tumor effects and biosafety at both the cellular and animal levels. This study proposed a new nanocatalytic therapeutic strategy called "magnetoelectric dynamic therapy", expanding the application prospects of magnetic-responsive nanomedicine materials in cancer therapy.

In the sensitization strategy of piezo-catalytic therapy, by integrating multiple mechanisms such as sonodynamic, photothermal, chemotherapy, and immune regulation, a promising paradigm for achieving enhanced efficacy and reduced toxicity for complex tumors is theoretically provided. However, most current research still remains at the level of simple functional superposition and phenomenological description. There is a lack of in-depth mechanistic explanation and quantitative evidence regarding whether true spatiotemporal synergy and mechanistic complementarity exist between different treatment modes. For example, will the local high temperature induced by the photothermal effect damage the crystal structure of piezoelectric materials, thereby weaken their catalytic activity? Could the spatiotemporal asynchrony between premature release of chemotherapeutic drugs and piezo-catalysis lead to synergistic failure? These fundamental questions have not been systematically answered. Future research urgently needs to move beyond simple phenotypic superposition from a "mechanism-driven" perspective, deeply investigate the intrinsic connections between energy conversion, material transport, and biological responses, and achieve precise synergy of different treatment modes in time, space, and dosage with the help of intelligent responsive material design and controllable triggering technology, thereby promoting synergistic therapy from "proof-of-concept" to "clinical utility."

### Tissue repair and regenerative medicine

Tissue engineering and regenerative medicine aim to repair or replace damaged tissues and organs 115, 116. Current strategies primarily revolve around cell therapy, tissue engineering, and bioactive molecules, often tightly integrated with nanotechnology to enhance therapeutic outcomes 117-119. For instance, delivering mRNA via lipid nanoparticles 120 or constructing smart materials 121 have all shown great potential in regenerative medicine 122. However, the field still faces challenges such as immunogenicity and delivery efficiency. Piezoelectric heterojunctions offer a unique solution: they can convert the body's own mechanical activities (e.g., walking stress, muscle contractions) into precise, endogenous electrical stimulation without the need for an external power source, thereby actively modulating cell behavior 123, 124. This "self-powering" characteristic makes them an ideal choice for constructing dynamically responsive tissue engineering scaffolds.

#### Bone tissue engineering

In the field of bone tissue engineering, the application strategies for piezoelectric heterojunctions are quite mature, and several reviews have systematically reported on their progress 125. The core principle is to promote bone regeneration and osseointegration by modulating the electrical microenvironment.

**Figure 6:**
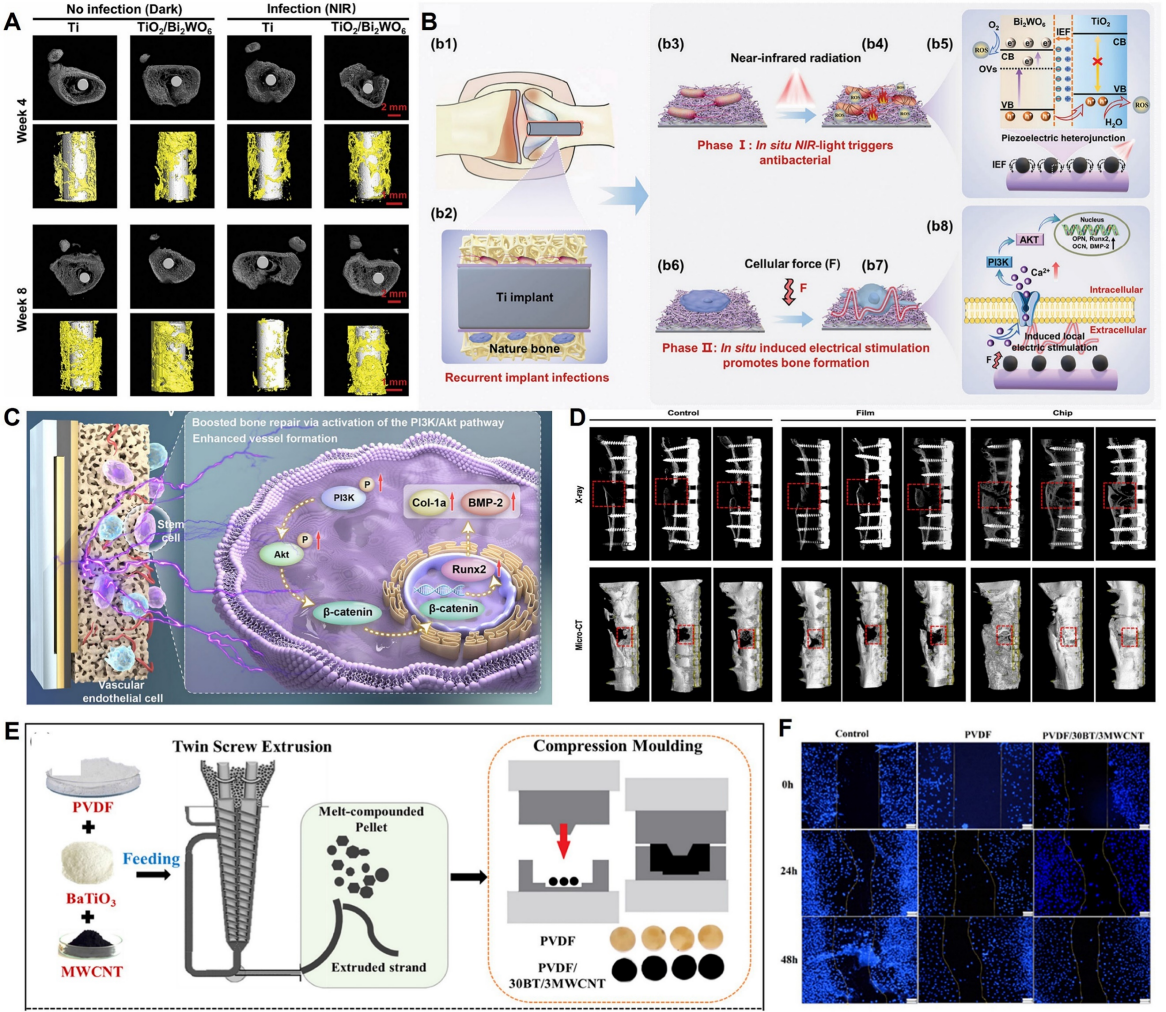
** Piezoelectric heterojunction for regenerative medicine. (A)** Micro-CT images showing the bone repair around different implants. Adapted with permission from 82, copyright 2024 The Author(s). **(B)** NIR light-triggered ROS (facilitated by the built-in electric field, IEF) and heat production for photodynamic and photothermal antibacterial therapy, and cellular force-induced electrical stimulation for bone formation. b1,b2) Implantation of TiO2/Bi2WO6 with bacterial infection in bone defect model. b3) Live bacteria grown on the surface of TiO2/Bi2WO6. b4) Bacterial death due to photothermal and photodynamic therapy. b5) Under NIR irradiation, oxygen vacancies (enabling the heterojunctions to absorb NIR light to produce heat for photothermal antibacterial therapy) areformedinBi2WO6 by using ethylene glycol to remove lattice oxygen ions, and IEF induces the separation of electron-hole pairs from Bi2WO6 to TiO2, which promotes the ROS production for photodynamic antibacterial therapy. b6) Growth of bone marrow mesenchymal stem cells (mBMSCs) on TiO_2_/Bi_2_WO_6_. b7) The electrical stimulation, generated by cellular force due to the presence of piezoelectric Bi2WO6, facilitates osteogenic differentiation of mBMSCs and thus effectively promotes osseointegration. b8) The force from mBMSCs generates a local electric field that promotes inward Ca2+ flux, which activates the PI3K-AKT signaling pathway, upregulates the expression of osteogenesis-related genes (OCN, OPN, Runx2, and BMP-2), and facilitates osteogenic differentiation. Adapted with permission from 82, copyright 2024 The Author(s). **(C)** The smart chip is a multilayer structure, the chip synergistically activates the PI3K/Akt signaling pathway, thereby promoting the proliferation and osteogenic differentiation of stem cells. Meanwhile, the chip enhances angiogenesis by upregulating VEGF-A and CD31 expression. Hence, the chip provides a promising strategy for critical-sized bone defect repair through robust electrical stimulation and vascularized bone regeneration, achieving an efficient scaffold-free bone repair. Adapted with permission from 127, copyright 2025 The Author(s). **(D)** 3D reconstruction images of bone defect in the control, film, and chip groups at 4 weeks post-operation. Adapted with permission from 127, copyright 2025 The Author(s).** (E)** Schematic diagram for PVDF composite preparation using micro-compounder followed by compression molding. Adapted with permission from 128, copyright 2023 Elsevier Ltd.** (F)** *In vitro* migration assays to evaluate the migratory capacity of MC3T3-E1 cells on different samples over 48 h of cell incubation. Adapted with permission from 128, copyright 2023 Elsevier Ltd.

One strategy is to construct multifunctional implant materials that synergistically provide antibacterial and bone-regenerative functions. For example, Fan *et al.* 82 developed a piezoelectric heterojunction implant material based on TiO₂/Bi₂WO₆. Under NIR irradiation, this material could efficiently kill bacteria (antibacterial rate >98%) through photothermal and photodynamic effects. Simultaneously, the mechanical forces exerted by growing stem cells (murine bone marrow-derived mesenchymal stem cells, mBMSCs) on the material's surface activated its piezoelectric effect, generating a local electric field (approx. 1-11 mV). This promoted intracellular calcium ion influx, activated the PI3K-AKT signaling pathway, and upregulated osteogenesis-related genes (OCN, OPN, Runx2, BMP-2), thereby significantly enhancing osseointegration and bone regeneration. *In vivo* experiments further confirmed that the material could effectively clear bacteria and promote new bone formation in an infected bone defect model **(Figure 6A-B)**. Another strategy is to mimic and maintain a physiological electrical microenvironment. Zhang *et al.* 126 constructed a flexible nanocomposite film containing PDA-modified BaTiO₃ nanoparticles and P(VDF-TrFE), which successfully mimicked the endogenous physiological potential (approx. -76.8 mV) and maintained long-term stability (retaining 56% after 12 weeks). In this study, they emphasized that restoring the physiological electrical microenvironment is a key strategy for enhancing bone regeneration. The underlying mechanism is that the polarized membrane surface forms a sustained electric field, which recruits bone marrow-derived mesenchymal stem cells (BM-MSCs) through galvanotaxis and promotes their adhesion, spreading, and osteogenic differentiation, ultimately leading to the rapid repair of critical-sized rat cranial defects.

Utilizing endogenous physiological vibrations to activate the piezoelectric effect is a frontier direction for achieving scaffold-free, dynamic bone repair. Li *et al.* 127 developed a self-enhancing piezoelectric chip based on aluminum nitride (AlN) that could generate stable and lasting electrical signals (current density up to 3.29 μA/cm²) solely from physiological vibrations (such as muscle contractions), without external stimulation or scaffold implantation. In a rabbit femoral defect model, the chip significantly promoted the osteogenic differentiation of BMSCs and angiogenesis (upregulating VEGF-A, CD31 expression) by activating the PI3K/Akt signaling pathway, achieving vascularized bone defect repair within 4 weeks **(Figure 6C-D)**. This chip can be integrated with clinical orthopedic bone plate systems, offering a "plug-and-play," scaffold-free bone repair strategy.

In-depth mechanistic studies on the interaction between piezoelectric composites and cells have provided a basis for rational design, with voltage-gated calcium channels (VGCCs) identified as key signal transduction nodes. The PVDF/BaTiO₃/MWCNT composite prepared by Bhaskar *et al.* 128 exhibited excellent blood compatibility and significantly promoted the adhesion, proliferation, and mineralization of pre-osteoblastic cells** (Figure 6E-F)**. Research by Chernozem *et al.* 129 showed that incorporating rGO into a PHB scaffold enhanced its piezoelectric response (d_vert=0.59 pm/V, d_lat=1.06 pm/V). The mechanism may involve the activation of VGCCs by the local electric field generated by the piezoelectric response, which in turn modulates signaling pathways like ERK1/2. The study by Vaidya *et al.* 130 further confirmed this pathway: the piezoelectric potential (up to 25 mV) from their PVDF-ZnO nanofiber coating also promoted Ca²⁺ influx by activating VGCCs, subsequently upregulating osteogenesis-related genes through the calcium-calmodulin signaling pathway.

#### Nerve and cartilage regeneration

**Figure 7:**
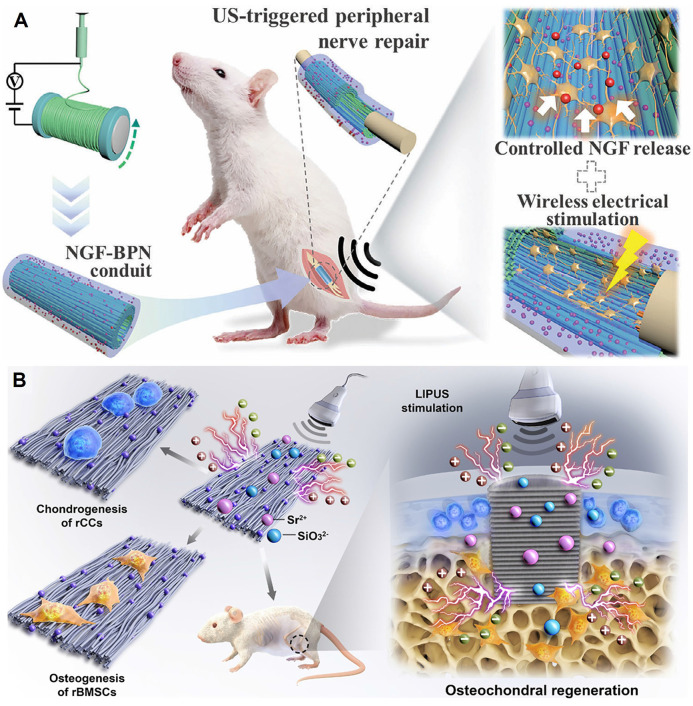
** Regulating cell behavior. (A)** Schematic diagram of the US-responsive aligned piezoelectric nanofibers derived hydrogel conduits for peripheral nerve regeneration. Adapted with permission from 87, copyright 2024 Wiley-VCH. **(B)** The sustained release of bioactive Sr and SiO_ 3_ ions, combined with the electrical charges generated by LIPUS stimulation, synergistically promotes chondrogenesis of rCCs and osteogenic differentiation of rBMSCs *in vitro*, thereby simultaneously enhancing cartilage regeneration and subchondral bone reconstruction *in vivo*. Adapted with permission from 133, copyright* 2025 Published by Elsevier Ltd*.

In nerve regeneration, piezoelectric heterojunctions can provide continuous, gentle electrical stimulation to guide the differentiation of neural stem cells and promote axon growth. For example, Xu *et al.* 83 developed an ultrasound-responsive piezoelectric nanofiber hydrogel nerve guidance conduit (NGC). Its inner layer consisted of aligned P(VDF-TrFE)/BTO electrospun nanofibers, and the outer layer was a thermosensitive hydrogel loaded with nerve growth factor (NGF). Under US, the inner layer generated a wireless electric field stimulus via the piezoelectric effect, promoting directional neurite extension. Simultaneously, the sonothermal effect caused the outer hydrogel to contract, achieving controlled release of NGF. This dual-pathway synergy of "electrical stimulation + neurotrophic factor release" significantly promoted the structural reconstruction and functional recovery of the rat sciatic nerve **(Figure 7A)**. Zhang *et al.* 131 developed a hydrogel system based on Fe₃O₄@BaTiO₃ magnetoelectric nanoparticles. By wirelessly activating the piezoelectric effect with an external pulsed magnetic field, they generated a surface potential change (up to 8 µV). This system significantly promoted neurite extension in PC12 cells and facilitated axon regeneration while inhibiting glial scarring in a spinal cord injury model. Mechanistic studies by Wang *et al.* 132 further revealed that a BTO/rGO piezoelectric nanopatch, under US stimulation, could cause Ca²⁺ influx by activating VGCCs. This, in turn, activated the CaMK II/CREB signaling pathway, upregulated BDNF expression, and significantly promoted the differentiation of neural stem cells into functional neurons.

In cartilage regeneration, the piezoelectric effect also shows unique advantages, often synergizing with other bioactive signals. Liu *et al.* 133 constructed a biodegradable pPLLA/SrSiO₃ composite scaffold that could simultaneously release Sr²⁺/SiO₃²⁻ bioactive ions and generate piezoelectric charges under low-intensity pulsed ultrasound (LIPUS) stimulation. These two signals synergistically activated the P2X1 purinergic receptor calcium channel, causing Ca²⁺ influx and jointly promoting the integrated regeneration of cartilage and subchondral bone **(Figure 7B)**. A nanocomposite hydrogel containing BTO and GO, developed by Ricotti *et al.* 134, significantly promoted the chondrogenic differentiation of hMSCs under US stimulation. The mechanism likely involves the piezoelectric micro-electric field (predicted to be up to ~43.1 µV) influencing the cytoskeleton, Wnt, and integrin signaling pathways through channels like VGCCs, thereby enhancing cartilage formation and inhibiting inflammation.

In general, piezoelectric materials hold immense potential in regenerative medicine. Current research is largely focused on low-dimensional piezoelectric materials, which are integrated into piezoelectric scaffolds, hydrogels, and nanocomposites to mimic the natural tissue microenvironment, promote cell differentiation and regeneration, enhance mechanotransduction, and provide self-powered electrical stimulation 135, 136. Upon investigation, we found that although many studies do not explicitly define them as such, they have widely applied strategies involving piezoelectric heterojunctions or composites. These strategies can be broadly categorized into several types: first, the synergistic enhancement between different piezoelectric materials (piezoelectric-piezoelectric type), such as combining inorganic ceramics with high piezoelectric coefficients with flexible piezoelectric polymers 126, 83, 137, 138; second, interfacial charge modulation with semiconductors (piezoelectric-semiconductor type) to promote charge separation or introduce other responsive properties 131, 139; and third, the coupling of effects with functional materials, for example, by combining with bioactive ceramics to achieve electro-chemical signal synergy 133 or compounding with carbon materials to regulate the conductivity and cytoaffinity of the microenvironment 134.

However, the field is still limited by a "black box" understanding of the underlying mechanisms. Most current research is content with observing the final cellular phenotype but lacks a detailed analysis of the quantitative structure-activity relationship between electrical signal parameters and the activation of specific intracellular signaling pathways. Gaining a deep understanding of the spatiotemporal process of the interaction between the piezoelectric micro-electric field and the cell membrane is the key to achieving rational design and promoting clinical translation in the future.

### Antibacterial and infection control

Antibiotic resistance has become a global health crisis, making the development of novel, non-antibiotic-dependent antibacterial strategies an urgent priority. Piezoelectric heterojunctions offer a highly promising solution through their unique "physical-chemical synergistic" bactericidal mechanism. Their core advantages are threefold: first, the ROS generated by piezocatalysis have broad-spectrum antibacterial properties, effective against both Gram-positive and Gram-negative bacteria; second, strategies using US as a driving source have high tissue penetration, enabling non-invasive treatment of deep-seated infections; finally, the electrical signals generated by the piezoelectric effect can synergistically promote the regeneration and healing of surrounding tissues, achieving a dual "antibacterial-regenerative" function 81, 140.

Achieving an integrated "antibacterial-regenerative" effect is the ideal strategy for treating infected tissue defects, and piezoelectric therapy exhibits unique systemic advantages in this area. For example, Roy *et al.* 141 significantly enhanced the piezoelectric response of bismuth oxyiodide nanosheets through defect engineering (iron ion doping) and used macrophage membrane camouflaging for bacterial targeting. This study, published in Small, systematically demonstrated how this nanoplatform, under US activation, could simultaneously address both superficial (methicillin-resistant Staphylococcus aureus (MRSA) skin infections) and deep-seated (osteomyelitis) challenges. Its core mechanism is multifaceted: first, the enhanced piezocatalytic effect generates a large amount of ROS to efficiently eliminate drug-resistant bacteria; second, the piezoelectric signal can reprogram pro-inflammatory M1 macrophages into anti-inflammatory and pro-reparative M2 types; more importantly, for the osteomyelitis model, the piezoelectric electrical stimulation can also directly promote the differentiation of mesenchymal stem cells into osteoblasts by activating calcium ion channels, thereby accelerating bone tissue regeneration. This work perfectly illustrates how a single piezoelectric platform can synergistically achieve the triple therapeutic goals of "bactericidal, anti-inflammatory, and pro-regenerative" effects. The antibacterial mechanism of piezoelectric heterojunctions is multifaceted, including synergistic ion toxicity in addition to the mainstream ROS-induced oxidative damage. For instance, the "herbal piezoelectric heterojunction" designed by Zhang *et al.* 142 utilizes the piezoelectric effect to promote the endocytosis of Cu²⁺ ions, inducing cuproptosis in bacteria.

In addition to treating deep-seated bone infections, the same research group has further extended this concept to the therapy of bacteria-infected burn wounds. In a recent study published in Nano Energy, Roy and Guo *et al.* designed a multifunctional, intelligent piezoelectric hydrogel 143. By co-doping BaTiO₃ with Cu²⁺ and Zn²⁺ ions, they achieved synergistic antibacterial effects through the combination of PZDT and CDT. The most pioneering aspect of this work lies in its dual-mode therapeutic strategy driven by two distinct forms of mechanical energy: initially, high-frequency external ultrasound is used to trigger PZDT, generating a burst of ROS for rapid bactericidal action; subsequently, during the healing phase, the hydrogel converts low-frequency mechanical deformations from daily physical activities into weak electrical signals. These signals serve to reprogram macrophages into the pro-reparative M2 phenotype. This study pioneeringly demonstrates the immense potential of using piezoelectric materials as 'self-powered' immunomodulators, paving the way for the development of next-generation 'smart dressings' that operate in synergy with bodily movements without the need for external equipment.

**Figure 8:**
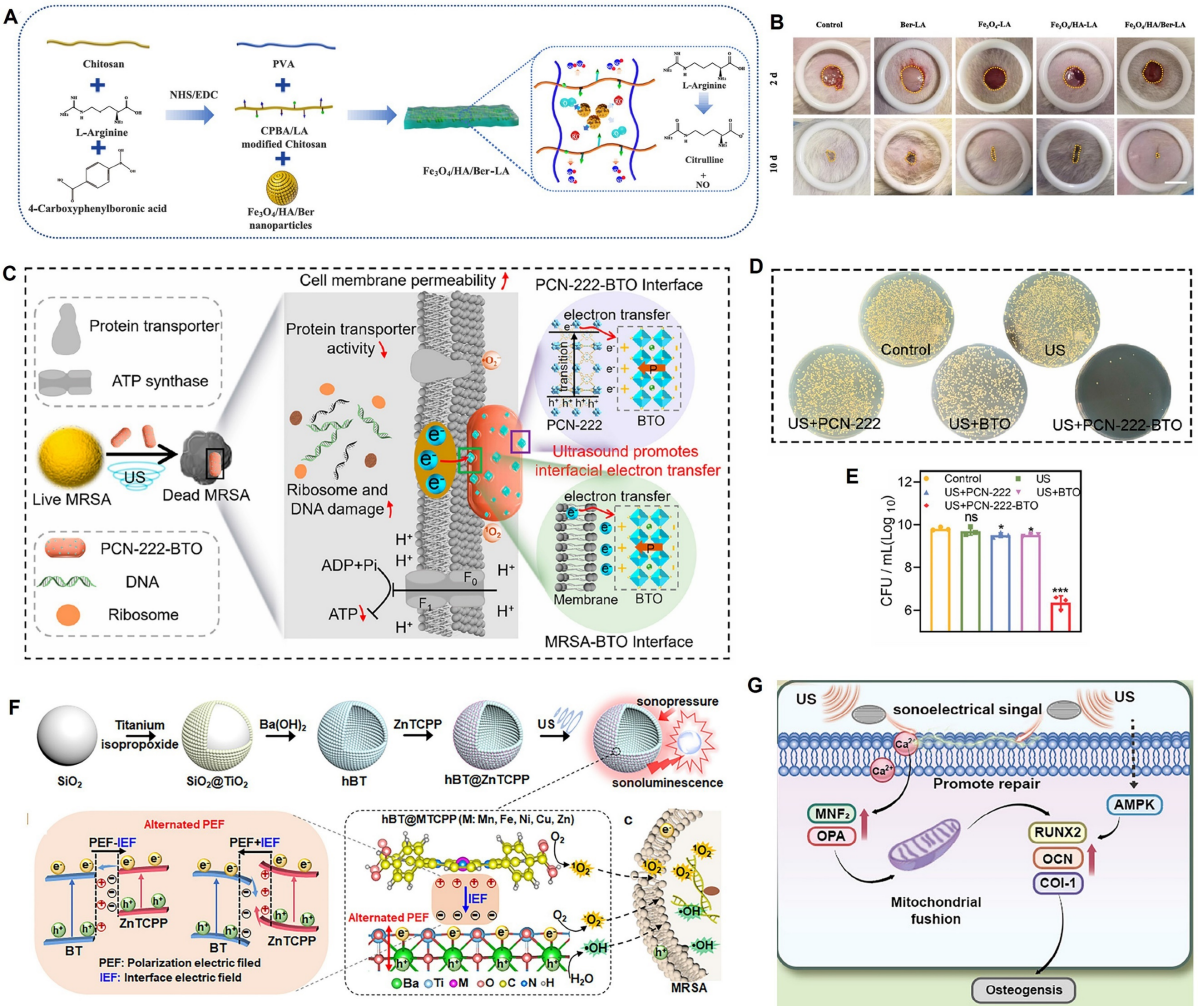
** Antibacterial and infection control. (A)** The synthesis route of Fe3O4/HA/Ber-LA hydrogel. Adapted with permission from 144, copyright 2024 Elsevier B.V. **(B)** representative macroscopic appearance of wounds (scale bar, 1 cm). Adapted with permission from 144, copyright 2024 Elsevier B.V. **(C)** Mechanism of ultrasound-promoted interfacial electron transfer. Adapted with permission from 145, copyright 2023 American Chemical Society.** (D)** and **(E)** Spread plate and Number of MRSA colonies after treatments with US, US+PCN-222, US+BTO, US+PCN-222-BTO, *p<0.05, ***p<0.001. Adapted with permission from 145, copyright 2023 American Chemical Society. **(F)** Schematic illustration of the preparation process and sonocatalytic mechanism of hBT@ZnTCPP heterojunction. Adapted with permission from 146, copyright 2025 Wiley-VCH GmbH. **(G)** Schematic illustration of ultrasound-active DTO/BTO for treating S. aureus-infected bone defect and promoting osteogenic differentiation. Adapted with permission from 147, copyright 2025 The Author(s).

In the construction of multifunctional antibacterial hydrogels, Wang *et al.* 144 developed a chitosan-based composite hydrogel (Fe₃O₄/HA/Ber-LA) that achieved immuno-sonodynamic therapy (ISDT) under US drive, significantly accelerating the healing of bacteria-infected wounds. In this hydrogel, Fe₃O₄ nanoparticles formed a heterojunction with berberine (Ber), which significantly reduced the bandgap, enhanced sonocatalytic activity, and promoted ROS generation. The ROS further catalyzed the grafted L-arginine (LA) to release nitric oxide (NO), which not only directly killed bacteria but also promoted the polarization of macrophages to the pro-inflammatory M1 type, enhancing their phagocytic ability. In the later stages of infection, the released berberine exerted anti-inflammatory and antioxidant effects, inducing macrophage conversion to the anti-inflammatory M2 type and promoting angiogenesis (upregulation of vascular endothelial growth factor (VEGF) secretion) and tissue repair. *In vitro* and *in vivo* experiments showed that the hydrogel had high bactericidal activity against S. aureus and E. coli under US (antibacterial rate >98%) and accelerated wound healing by modulating the immune microenvironment and promoting collagen deposition** (Figure 8A-B)**. To enhance interfacial charge transfer for improved antibacterial efficiency, Yu *et al.* 145 designed a US-activated, piezoelectric-responsive heterojunction material, PCN-222-BTO. By promoting electron transfer at both the abiotic interface (between PCN-222 and BTO) and the bio-abiotic interface (between bacteria and BTO), it achieved highly efficient sonodynamic sterilization. In *in vitro* experiments, the material achieved a bactericidal efficiency of up to 99.96% against MRSA and 99.77% against Escherichia coli (E. coli) after just 15 minutes of US treatment. Its antibacterial mechanism includes BTO polarization under US to generate a BIEF, which promotes the transfer of excited electrons from PCN-222 to BTO, significantly increasing ROS (especially ¹O₂) generation. Simultaneously, BTO extracts electrons from the bacterial membrane, interfering with the bacterial respiratory chain and leading to membrane potential depolarization, adenosine triphosphate (ATP) synthesis inhibition, and ribosome and DNA damage. In a rat model of MRSA-infected osteomyelitis, PCN-222-BTO combined with US treatment significantly reduced infection and promoted bone repair, with efficacy superior to the clinical antibiotic vancomycin, demonstrating good biocompatibility and therapeutic potential **(Figure 8C-E)**.

Constructing an advanced S-scheme heterojunction is another effective enhancement strategy. Wei *et al.* 146 built an S-scheme heterojunction material, hBT@ZnTCPP, by combining hollow barium titanate (hBT) with zinc-coordinated tetrakis(4-carboxyphenyl)porphyrin (ZnTCPP). US triggered the piezoelectric effect to enhance the interfacial electric field (IEF), significantly increasing the generation efficiency of sonocatalytic ROS. Experimental results showed that at a concentration of 50 µg/mL, just 5 minutes of US treatment could achieve highly efficient killing of planktonic MRSA and Pseudomonas aeruginosa, with a sterilization rate of 99.8%. The effect on biofilm removal was also significant, with only 0.8% biofilm residue at a concentration of 100 µg/mL. The antibacterial mechanism was mainly attributed to the enhanced IEF promoting electron-hole separation, generating a large amount of ROS (especially ·OH and ¹O₂), which led to bacterial membrane depolarization, structural damage, and intracellular oxidative stress. It also synergistically achieved efficient and rapid antibacterial action by downregulating bacterial genes related to ribosome synthesis, transmembrane transport, and biofilm formation** (Figure 8F)**. Defect engineering has also been proven to be key to enhancing piezoelectric antibacterial performance. Wang *et al.* 147 prepared a heterojunction material (DTO/BTO) of defective TiO₂ (DTO) with OVs and enriched Ti³⁺ species and BTO using laser cladding technology. After 20 minutes of US treatment, DTO/BTO achieved a bactericidal efficiency of up to 99.83% against S. aureus and effectively inhibited biofilm formation. Its antibacterial mechanism primarily stemmed from the massive generation of US-induced ROS (such as ·OH and ¹O₂), leading to bacterial membrane rupture, protein leakage, respiratory chain disruption, and GSH depletion, which triggered intense oxidative stress. Furthermore, DTO/BTO had good biocompatibility and could promote osteogenic differentiation by upregulating mitochondrial fusion genes **(Figure 8G),** demonstrating significant antibacterial and osseointegration capabilities in a rat model of infected bone defects and providing an efficient antibiotic-alternative treatment strategy for deep tissue infections.

Piezoelectric heterojunctions demonstrate a "double-edged sword" effect in the antibacterial field through the synergy of physical puncture and chemical oxidation, offering a highly promising alternative strategy to overcome antibiotic resistance. However, their translational application is facing severe challenges moving from idealized experimental environments to complex real biological environments. The >99% bactericidal rates reported in current studies mostly originate from the short-term effects of high-concentration materials and high-intensity US in *in vitro* planktonic bacteria models 148-152. This creates a huge gap compared to the extreme conditions they might encounter in *in vivo* infection microenvironments, such as biofilm barriers, protein corona formation, and ROS depletion by the inflammatory microenvironment. More critically, most studies separate "sterilization" from "promoting healing," failing to address the core issue of how the large amount of bacterial debris and inflammatory factors produced after efficient sterilization can be effectively cleared, and whether the electrical signals generated by piezoelectric materials can actively switch to an immune regulation and tissue regeneration mode after sterilization.

### Biosensing and self-powered theranostic systems

By virtue of their unique mechano-electrical conversion properties, piezoelectric heterojunctions are not only efficient therapeutic tools but also highly sensitive sensing elements. This dual capability gives them enormous potential in biosensing and in the construction of self-powered theranostic systems 153, 154.

In high-sensitivity biosensing, piezoelectric heterojunctions primarily achieve trace detection of biomarkers by enhancing the signal response. Liao *et al.* 155 synthesized urchin-like AuPt@BaTiO₃ microspheres as oxidase mimetics. They utilized the synergistic effect of the photo-piezoelectric effect and LSPR to significantly enhance the oxidase-like activity. Under the combined action of light and US, the material could efficiently promote the generation of superoxide anions (O₂•⁻), thereby rapidly oxidizing the chromogenic substrate TMB. Based on the inhibitory effect of GSH on this oxidation reaction, they constructed a high-sensitivity colorimetric detection platform. The platform could complete detection within 3 minutes, with a limit of detection as low as 0.225 µM and a linear range of 0.5-50 µM. It also demonstrated excellent recovery rates (99.91%-101.8%) and anti-interference ability in real serum samples. This study provides a new strategy for designing efficient nanozymes using the photo-piezoelectric effect, with potential applications in biosensing and clinical diagnostics. Wang *et al.* 156 pioneered the integration of the piezotronic effect with photoelectrochemical biosensing and a self-powered chemical amplification strategy to construct a high-performance detection platform for exosomal miRNA based on a Bi₂WO₆/Cu₂S heterojunction. Its core feature is a coupled sensitization strategy that combines "physical field enhancement" with "chemical reaction amplification." On one hand, the piezoelectric effect (e.g., from US) was introduced to generate a polarization field in Bi₂WO₆, which physically drove the efficient separation of photo-generated charges and greatly enhanced the initial photocurrent response. On the other hand, they innovatively designed a target-responsive MOF@CaO₂ nanoprobe that could self-supply H₂O₂ as an electron donor upon recognizing the target miRNA. This further suppressed charge recombination by consuming holes, achieving a secondary chemical amplification of the signal. This piezoelectric-assisted, self-supplying electron donor mechanism not only fundamentally solved the critical bottleneck of severe charge recombination in PEC sensing, enabling ultra-high sensitivity (0.1 fM-1 µM) and low-cost detection, but also provided a paradigm-shifting approach for developing novel PEC bioanalysis enhanced by external field coupling. Meanwhile, heterojunctions themselves, even without the piezoelectric effect, play a key role in PEC sensing. Chen *et al.* 84 constructed a PEC biosensor based on a Z-scheme Bi₂O₃/CuBi₂O₄ heterojunction for the high-sensitivity and high-selectivity detection of aflatoxin B1 (AFB1). Synthesized via a one-step method, the Bi₂O₃/CuBi₂O₄ heterojunction, whose Z-scheme electron transport pathway was verified by density functional theory (DFT), exhibited enhanced visible light absorption and efficient separation of photo-generated carriers. The developed PEC biosensor showed excellent performance in AFB1 detection, with a limit of detection as low as 297.4 fg/mL and a wide linear range of 1.4 pg/mL-280 ng/mL. Its high recovery rate (93-112%), good repeatability, reproducibility, stability, and anti-interference ability were validated in real samples such as urine, lake water, peanuts, and wheat. These studies collectively demonstrate the great potential of heterojunctions to enhance chemical sensing signals by promoting charge separation.

**Figure 9:**
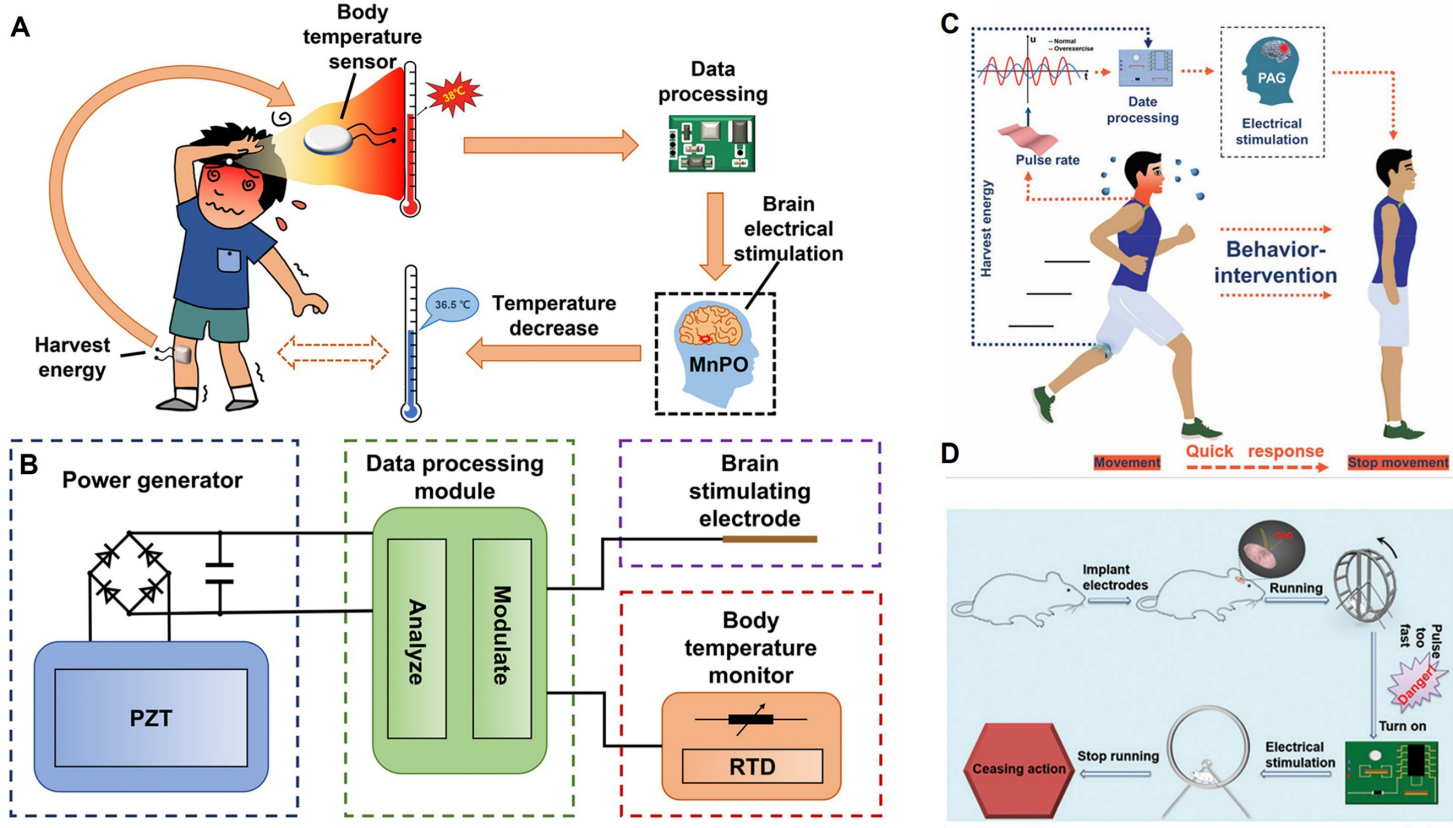
** Biosensing and self-powered theranostic systems. (A)** The self-powered wearable brain-machine-interface system for real-time monitoring and regulating body tempera ture. Adapted with permission from 158, copyright 2022 RSC Pub. **(B)** Four components of the brain-machine-interface system. Adapted with permission from 158, copyright 2022 RSC Pub. **(C)** Self-powered wearable brain-machine-interface system for the behavioral intervention of ceasing action. Adapted with permission from 159, copyright 2022 RSC Pub. **(D)** The experimental design of the brain stimulation in mice for the ceasing action. Adapted with permission from 159, copyright 2022 RSC Pub.

In self-powered wearable/implantable devices, piezoelectric heterojunctions can efficiently convert weak mechanical energy from human motion into electrical energy, providing continuous power for bioelectronic devices. This is particularly crucial in emerging fields like brain-computer interfaces (BCIs). For example, Wang *et al.* 157 constructed heterostructures by creating lattice-matched inorganic-inorganic core-shell structures (such as CoFe₂O₄-BaTiO₃ and NiFe₂O₄-BaTiO₃). They used a scanning tunneling microscope nanoprobe technique to measure the magnetoelectric coefficient at the single-nanoparticle level, avoiding the charge leakage issues common in traditional powder measurements. Ultimately, they achieved giant magnetoelectric coefficients of up to 5 V cm⁻¹ Oe⁻¹ (cobalt ferrite-based) and 2 V cm⁻¹ Oe⁻¹ (nickel ferrite-based) in 20 nm core-shell particles, an order of magnitude higher than previously reported 0-3 type composite particles. This breakthrough provides a key material basis for wireless brain-machine interfaces: by controlling the electrical polarization state of the nanoparticles with an external magnetic field, remote and precise neural stimulation or signal reading can be achieved. This holds revolutionary potential, especially in treating neurodegenerative diseases, brain tumors, and achieving human-machine intelligent interaction. Xu *et al.* 158 used a commercial lead PZT piezoelectric bimorph as the energy harvesting unit. This structure is essentially a piezoelectric heterojunction composed of two stacked PZT layers, which can generate a voltage output under external mechanical deformation (such as bending caused by human motion). The piezoelectric unit has a high piezoelectric constant (d₃₃=6.2×10⁻¹⁰ m V⁻¹) and good mechanical stability, allowing it to convert mechanical energy from daily human activities (like walking and running) into electrical energy. This energy powers the entire system, including a temperature detection unit, a data processing module, and a brain electrostimulator. The study demonstrated the energy harvesting performance of the piezoelectric heterojunction on the arm and knee, with a maximum output voltage of 23.16 V, which could quickly charge a capacitor to make the system completely self-powered **(Figure 9A-B)**. This design not only solves the power supply problem for wearable BCI systems but also, through the efficient energy conversion of the piezoelectric heterojunction, promotes real-time monitoring and regulatory treatment of diseases like heatstroke, expanding the practical application prospects of piezoelectric materials in medical-grade BCI systems. Similarly, Xiang *et al.* 159 used a PZT bimorph as an energy harvesting unit. This structure, through human motion (such as knee bending), could generate electrical energy with a maximum output voltage of 16 V and charge a 150 µF capacitor, powering the entire system (including a signal processing module and brain stimulation electrodes). This design showcases the advantages of piezoelectric heterojunctions in efficient energy harvesting and their potential in self-powered, wearable BCI systems for real-time physiological monitoring and neural regulation, providing a new technological path for future behavioral interventions and disease treatment **(Figure 9C-D)**.

More importantly, piezoelectric heterojunctions open up a new path toward achieving a true "closed-loop theranostic system," which perfectly aligns with the core concept of the journal Theranostics. In this framework, the piezoelectric material plays the dual role of a "diagnostician" and a "therapeutic agent."

On one hand, many piezoelectric heterojunctions inherently integrate imaging functionalities, which can be used for precise pre-treatment diagnosis and guidance. For example, the La-BFO nanozyme designed by Li *et al.* 74 possesses CT/MRI dual-modal imaging capabilities due to its constituent Bi and Fe elements. This allows for real-time tracking of the material's *in vivo* distribution to guide subsequent US therapy. Similarly, the BTAP nanoplatform constructed by Xu *et al.* 111 achieved CT imaging functionality by utilizing Au and Ba elements. This strategy of "imaging-guided therapy" is a prerequisite for achieving precision medicine.

On the other hand, the reversibility of the piezoelectric effect endows it with the potential for therapeutic efficacy monitoring. During treatment, changes in the lesion tissue (such as altered stiffness due to apoptosis, restoration of blood flow, etc.) can, in turn, affect the mechanical stress experienced by the piezoelectric material, thereby changing its electrical signal output. In theory, by interpreting these feedback electrical signals, it may be possible to achieve real-time, non-invasive monitoring of the therapeutic effect and dynamically adjust the treatment plan based on this feedback. Although this field is still in the proof-of-concept stage, constructing an integrated piezoelectric theranostic platform that combines "imaging-guided, on-demand therapy with efficacy monitoring" is a key future direction for the field.

## Challenges in clinical translation and future perspectives

As a cutting-edge interdisciplinary field in biomedical engineering, piezoelectric heterojunction technology is driving a revolutionary transformation in diagnostic and therapeutic models. However, it faces multiple challenges in biomedical applications that hinder its translation from laboratory research to clinical use. Deeply analyzing these challenges and identifying solutions is key to advancing the field.

### Biocompatibility, toxicology, and long-term safety

The foremost prerequisite for the clinical application of piezoelectric heterojunctions is strict adherence to biocompatibility requirements. Currently, high-performance piezoelectric ceramics represented by PZT are inherently biotoxic due to their lead content, which fundamentally limits their long-term *in vivo* applications 160. Therefore, developing high-performance lead-free piezoelectric materials (such as BaTiO₃, ZnO, KNN, and their derivatives 18) is a critical problem that the field must solve. Furthermore, the dynamic biological behavior of these materials *in vivo* must be thoroughly evaluated. After nanoparticles enter a physiological environment, their surfaces interact with biomolecules to form a "protein corona," a process that can alter their biological properties and targeting efficiency 117, 122. To comprehensively assess these dynamic processes and the potential toxicity of material degradation products, a systematic regulatory framework must be followed. Past experiences in tissue engineering and regenerative medicine have shown that a lack of strict control over starting materials and production processes is a major factor leading to clinical translation failure 115. Consequently, all piezoelectric heterojunctions intended for clinical application must comply with the International Organization for Standardization (ISO) 10993 standards and undergo comprehensive toxicological evaluation under Good Laboratory Practice (GLP) guidelines. The importance of this requirement is particularly prominent today. As relevant commentaries have pointed out, the field of regenerative medicine faces an "evidence crisis" due to inadequate regulatory procedures; establishing a robust, transparent, and evidence-based evaluation system is essential to ensure patient safety 118. To address the safety of long-term implants, a key research direction is material innovation. Recent progress in fully biodegradable, high-performance molecular ferroelectric crystals 161 provides a new technological path for developing transient implantable devices.

### Scalable fabrication and performance stability

The clinical application of piezoelectric heterojunctions requires that their fabrication processes can be scaled up from the laboratory to industrial production. This scale-up process presents technological challenges. Different types of heterojunctions require specific fabrication processes. For example, MBE can produce high-quality epitaxial heterojunctions, but its high cost and low throughput limit its large-scale application. Therefore, researchers are also exploring methods like impregnation-spin-coating to prepare continuous piezoelectric films on flexible substrates, which offers a possibility for low-cost, large-scale production 18.

However, a core issue is how to ensure the stability of material performance and batch-to-batch consistency in large-scale production. This challenge stems from the control of the material's microstructure. Research has shown that piezoelectric performance is highly sensitive to atomic-level defects. For instance, the work by Duddella *et al.* confirmed that the density and spatial distribution of intrinsic point defects (such as CuZn antisite defects) in ZnO microwires directly determine their piezoelectric output voltage 162. This implies that in scalable production, fluctuations in raw material purity, growth temperature, or atmosphere could lead to changes in defect types or quantities, causing variations in product performance and thus affecting batch-to-batch consistency.

Moreover, different material systems have specific process limitations. A recent review pointed out that several commonly used materials face fabrication difficulties: the organic polymer P(VDF-TrFE) has stability issues and "demanding preparation processes," while inorganic crystals like boron nitride involve "complex and challenging preparation processes" 18. Therefore, the research focus in this field must shift from exploring new structures to establishing stable, repeatable, and scalable fabrication processes. This requires the development of standard operating procedures and quality control systems, which are fundamental requirements for achieving clinical translation.

### Comparison with other therapies

The future prospects of any emerging therapy depend on its unique value relative to existing technologies. To clearly position piezoelectric heterojunction therapy among the many advanced diagnostic and therapeutic modalities, a systematic, side-by-side comparison is essential. Table 5 provides such a comparative analysis, evaluating it against mainstream therapies like photodynamic therapy (PDT), SDT, PTT, and CDT from multiple dimensions, including energy source, tissue penetration depth, core mechanism, and major pros and cons.

The comparison in Table 5 clearly shows that the core advantages of piezoelectric heterojunction therapy lie in its complete independence from external light sources and its use of high-penetration mechanical energy (such as ultrasound) as a driving source. This characteristic gives it irreplaceable potential in treating deep-seated solid tumors (like pancreatic cancer and glioblastoma) and orthopedic infections, areas where traditional light-based therapies are difficult to apply effectively. Compared to SDT, which also relies on ultrasound, piezoelectric materials serve the dual role of sonosensitizer and energy converter, simplifying the system design. Furthermore, the multifunctional integration capability of piezoelectric heterojunctions allows them to be easily combined with other therapies (such as magnetothermal therapy or chemotherapy) to produce significant synergistic enhancement. However, it must also be recognized that the current energy conversion efficiency of piezoelectric therapy (from mechanical to chemical energy) is relatively low, and its complex bio-material interaction mechanisms are yet to be fully elucidated. Future research must focus on improving its therapeutic efficiency and biosafety through novel material design and mechanistic exploration, in order to truly leverage its unique advantages in deep-tissue diagnostics and therapeutics.

### Future directions

Although piezoelectric heterojunctions have demonstrated significant application potential in the biomedical field, achieving true clinical translation requires future research to focus not only on enhancing efficacy but also on the precise regulation and safety of the therapeutic process. Recently, a highly inspiring new direction is the "ROS Balancing" strategy. This strategy aims to upgrade piezoelectric materials from simple "ROS generators" to "ROS levers" that can dynamically regulate oxidative stress levels—that is, efficiently generating ROS to kill lesions during treatment, and then actively scavenging excess ROS to protect normal tissues after treatment. As a recent review systematically elaborated, it is possible to achieve intelligent regulation of ROS levels through various strategies such as constructing heterojunctions, defect engineering, mitochondrial targeting, or hydrogen production 163. This concept places higher demands on the future design of piezoelectric heterojunctions and also presents deeper scientific challenges. To make the leap from empirical exploration to rational design, future research needs to advance synergistically in mechanistic elucidation, material innovation, and system integration.

Currently, the most central challenge in the field is the insufficient understanding of the fundamental interaction mechanisms between piezoelectric heterojunctions and biological systems. Specifically, several key scientific questions need to be addressed: First, in the complex physiological environment, the dynamic processes of charge separation and migration at the heterojunction interface and their interaction with biomolecules are still unclear, lacking in-situ, real-time characterization techniques at the nanoscale. Second, the molecular transduction mechanisms by which cells precisely perceive the electrical signals generated by the piezoelectric effect and convert them into specific cellular biological effects (such as ion channel regulation, signaling pathway activation) remain to be elucidated. Furthermore, establishing a comprehensive theoretical model that can accurately describe the coupling of multi-physics fields (mechano-electro-chemo-bio) across spatial scales (from molecule to tissue) and temporal scales (from nanoseconds to months) remains a major difficulty in current research 164. Solving these fundamental scientific problems is a prerequisite for achieving precise prediction and controllable regulation of piezoelectric biological effects.

To address the above challenges, future research needs to focus on mechanism-driven material innovation, deeply integrated with advanced computational methods. The development of new materials should go beyond merely pursuing enhanced piezoelectric performance and must serve the dual goals of biocompatibility and functional tunability. On one hand, there is a continuing need to develop high-performance lead-free and all-organic piezoelectric materials, such as bio-based materials self-assembled from amino acids or short peptides, which provide a platform for precisely tuning piezoelectric properties and biological functions through molecular engineering 18, 165. On the other hand, porous framework materials with regular channels and designable structures (such as MOFs and covalent-organic frameworks (COFs)) 166, 167 offer ideal carriers for constructing multifunctional composite systems. Faced with such a vast material design space and complex multi-field coupling processes, the deep integration of multi-scale simulations and artificial intelligence technology is an inevitable choice to accelerate the R&D process. Through high-throughput computing and machine learning algorithms, quantitative relationship models between material "structure-performance-bioeffect" can be constructed, enabling the inverse design and performance prediction of novel piezoelectric heterojunctions. At the same time, combining multi-physics coupling simulations with experimental characterization can deeply reveal the working mechanisms of piezoelectric heterojunctions in specific biological microenvironments, providing theoretical guidance for experimental design and validating scientific hypotheses 168.

Furthermore, achieving high spatiotemporal precision and ensuring therapeutic safety are prerequisites for clinical translation. For deep-tissue applications, focused ultrasound (FUS) has become a key enabling technology. FUS can non-invasively deliver mechanical energy to a millimeter-sized focal region deep within the body, thereby achieving precise activation of piezoelectric heterojunctions only within the target lesion. This maximizes the therapeutic effect while minimizing off-target effects 169. In terms of safety, a significant advantage of piezocatalytic therapy over photothermal therapy or high-intensity focused ultrasound (HIFU) ablation is its minimal thermal effect. Under the typical low-intensity ultrasound conditions used for activation (usually < 3 W/cm^2^), the temperature rise is negligible, thus effectively avoiding thermal damage to surrounding healthy tissues, a point that has been confirmed in many cutting-edge studies 12. Nevertheless, strict monitoring of local temperature and long-term biological effects remains a necessary step in preclinical and clinical evaluations.

## Conclusion

Through rational interface engineering, piezoelectric heterojunctions have become a powerful platform technology in the biomedical field. However, the field is at a critical turning point, shifting from "phenomenon-driven" discovery to "mechanism-driven" design. Currently, the main obstacle hindering its clinical translation is still the insufficient understanding of the complex "mechano-electro-chemo-bio" multi-field coupling mechanisms at the bio-material interface.

Future breakthroughs will depend on the synergistic development of three pillars: (1) elucidation of fundamental mechanisms, by using in-situ characterization and multi-scale simulations to visualize dynamic interfacial processes; (2) acceleration of material innovation, by utilizing artificial intelligence and high-throughput screening to rationally design biocompatible, biodegradable, and intelligent piezoelectric systems; and (3) integration of intelligent systems. The latter aims to evolve piezoelectric heterojunctions from single-function therapeutic tools into implantable or wearable intelligent theranostic platforms. Such systems will integrate self-powered sensing and computing units to monitor signals from the lesion microenvironment in real-time and adaptively regulate the therapeutic output, ultimately achieving "sense-analyze-respond" closed-loop precision medicine.

Ultimately, these concerted efforts will propel piezoelectric heterojunction technology from a novel class of material tools into a core enabling technology that provides revolutionary solutions for major health challenges such as cancer, tissue regeneration, and chronic diseases.

## Figures and Tables

**Table 1:** Classification of piezoelectric heterojunctions by material dimensionality

Classification	Schematic structure	Core advantages	Key challenges	Typical preparation methods	Potential biomedical applications
0D-2D	Nanodots attached to sheets	High specific surface area, inhibits agglomeration, conductive channels	Uniform distribution control	Hydrothermal, in-situ growth, self-assembly	Nano-sensitizers (therapy)
1D-2D	NWs/NRs intertwined with sheets	Efficient stress transfer, 3D charge network	Orientation and density control	CVD growth, transfer printing	Flexible/implantable sensors
2D-2D (vdW)	2D layered stacking	Clean interface, tunable band gap, external sensitivity	Large-scale prep. & transfer	Mechanical exfoliation, CVD, wet transfer	Wearable electronics, micro/nano devices
3D-2D	2D material covers 3D surface	Strong piezoelectricity + surface functionalization	Interface adhesion & stability	Transfer, coating, growth	High-performance composite devices
3D-3D	Multilayer thin film structure	Mature process, easy integration	Lattice mismatch & stress	Sputtering, PLD, ALD	MEMS biosensors

**Table 2:** Classification of piezoelectric heterojunctions by material composition and properties

Classification	Core mechanism	Typical material systems	Advantages	Challenges	Biomedical application focus
Piezo-Semiconductor-Semiconductor	Forms p-n junction, BIEF & piezoelectric field synergy	n-ZnO/p-Si, n-TiO₂/p-NiO	High charge sep. efficiency, tunable band gap	Lattice/thermal mismatch, interface states	Self-powered biosensing
Piezoelectric-Semiconductor	Strong piezo-potential modulates semiconductor channel (Piezo-electronic effect)	PZT/MoS₂, PMN-PT/GaN	Strong modulation capability, large output signal	Biocompatibility, interface compatibility	High-sensitivity mechanical sensing
Piezoelectric-Piezoelectric	Piezoelectric effect superposition & coupling, performance synergy	ZnO/AlN, PVDF/BaTiO₃	Performance enhancement, broadened bandwidth, good flexibility	Polarization matching, stress transfer	Flexible wearable devices
Piezoelectric-Metal	Forms Schottky junction, modulates barrier; efficient charge collection	ZnO NW/Au, BaTiO₃@Au NPs	Simple structure, high charge collection efficiency	Interface fatigue, long-term stability	Basic sensing, catalytic therapy

**Table 3:** Quantitative comparison of catalytic performance for representative piezoelectric heterojunctions

Heterojunction system	Structure type	Piezoelectric properties	Stimulation conditions (US)	ROS generation	Catalytic efficiency	Application
BTO/MoS₂@CA 15	Core-shell, 0D/2D	Typical butterfly curve	1.0 MHz, 1.5 W cm⁻²	Strongest ·OH signal	POD-like activity: 76.36 U/mg	Tumor pyroptosis therapy
Ov-BOS 73	Interlayered Self-Heterojunction	d*₃₃ = 203 pm/V	1.0 MHz, 1.0 W cm⁻²	Detected ¹O₂, ·OH, ·O₂⁻	Higher MB degradation rate vs BFO	Tumor pyroptosis therapy
Fe-SAs@PCN 76	0D/2D, Single-atom	d*₃₃ ≈ 5.8 pm/V	40 kHz, 50 W	Enhanced ROS yield vs MoS₂	Significant GSH depletion	Cuproptosis/Ferroptosis synergy
La-BFO 74	Doped piezocatalyst	Typical butterfly curve	1.5 W cm⁻²	Superior to ZIF-8	Significant GSH depletion	SDT & Cuproptosis synergy
Bi₂MoO₆/PB-Au 78	Ternary Heterojunction	d₃₃ = 20.3 pm/V	1.0 MHz, 1.0 W cm⁻²	4.8-fold ·OH yield vs PCN	~91% RhB degradation in 120 min	Piezocatalysis-CDT synergy
BiCuSeO NSs 80	2D Natural Superlattice	Typical butterfly curve	NIR-II (PTE) & US (Piezo)	Strongest ·OH signal in ESR	Highest MB degradation	Tumor ferroptosis therapy
Fe-MoS₂ 12	2D Nanosheet w/ single-atom	d₃₃ ≈ 9.22 pm V⁻¹	1.0 MHz, 1.0 W cm⁻²	¹O₂ gen. (60.22% DPA degradation)	GSH depletion & O₂ production	Hypoxia-relieved SDT
Cu-based2D MOF 75	2D MOF	Typical butterfly curve	1.0 W cm⁻², 4 min	Detected ·OH and ·O₂⁻	Significant MB degradation	Tumor therapy
Au@BaTiO₃ 77	Schottky junction	Tetragonal phase (XRD)	1.0 MHz, 1.5 W cm⁻²	Detected ¹O₂, ·O₂⁻, ·OH	Complete eradication of bacteria	PTE antibacterial & Piezo anticancer
CoFe₂O₄@BiFeO₃ 79	Core-shell, Magnetostrictive	Typical butterfly curve	AMF (1.6 mT)	2.66-fold ·OH yield vs BTO	>99% antibacterial rate (E. coli & S. aureus)	Antibacterial & Wound healing
TiO₂/Bi₂WO₆ 82	1D/2D Heterojunction	d₃₃: 8.122 pm V⁻¹	NIR & Cellular force	Highest DCFH-DA signal	>98% antibacterial rate	Antibacterial & Bone regen

**Table 4:** Guideline for selecting piezoelectric heterojunctions for specific biomedical applications

Application scenario	Key clinical challenges and requirements	Recommended heterojunction strategy	Rationale / Design principle	Representative system
Deep-tissue tumor therapy	Low penetration depth of stimuli; Hypoxia & high GSH in TME	Type-II or Z-scheme heterojunctions responsive to deep-penetrating energy (US, magnetic field). Integrate with O₂-generating/GSH-depleting components.	Maximize charge separation for high ROS yield. Overcome biological barriers to enhance therapeutic efficacy. US/magnetic field ensures deep energy delivery.	BMO/PB-Au 78; Fe-MoS₂ 12
Infected bone defect repair	Bacterial infection hinders regeneration; Need for simultaneous antibacterial and osteogenic effects.	Design multi-functional heterojunctions with both piezocatalytic antibacterial and piezo-electrical osteogenic activities.	Utilize ROS for broad-spectrum bacterial killing. Utilize sustained piezoelectric potential to mimic endogenous electrical cues and promote osteoblast differentiation.	TiO₂/Bi₂WO₆ 82
Nerve regeneration	Slow axonal growth; Need for directional guidance and sustained neurotrophic stimulation.	Fabricate aligned nanofiber-based piezoelectric scaffolds. Integrate with controlled release of growth factors.	Aligned topography provides physical guidance for axonal extension. Piezoelectric stimulation promotes NSC differentiation and neurite outgrowth.	P(VDF-TrFE)/BTNP nanofibers in hydrogel 61,83;
Antibacterial surface coating	Implant-associated infections; Biofilm formation.	Construct robust and stable heterojunction coatings (e.g., core-shell) on implant surfaces.	Provide on-demand, non-leaching antibacterial action upon mechanical stimulation (e.g., body movement, US cleaning), preventing biofilm formation without systemic antibiotic release.	Au@BaTiO₃ 77
Wearable health monitoring	Need for self-powered, flexible, and sensitive sensors.	Develop flexible polymer-based heterojunctions (e.g., PVDF composites) with high piezoelectric output.	Conform to skin and detect subtle physiological pressures (pulse, respiration). Convert mechanical energy into readable electrical signals without external power.	PVDF/BTO 67,68/ZnO^130^

**Table 5:** Comparative analysis of piezo-catalytic therapy with other advanced therapies 73,78,80,109,110.

Feature	Piezo-catalytic therapy	Photodynamic therapy	Sonodynamic therapy	Photothermal therapy
Energy source	Mechanical (US, movement)	Light (Vis-NIR)	Ultrasound	Light (NIR)
Penetration depth	Deep	Shallow	Deep	Limited
Primary mechanism	ROS generation, Ferroptosis, Cuproptosis, etc.	ROS generation (mainly ¹O₂)	ROS generation, Cavitation effects	Hyperthermia (>42 °C)
Key advantages	Deep tissue penetration Non-invasive Independent of external light Diverse mechanisms	High spatiotemporal precision Minimal thermal damage	Deep tissue penetration Non-invasive	High ablation efficiency Rapid therapeutic effect
Key limitations	Relatively lower energy conversion efficiency Complex mechanism Potential material toxicity	Limited penetration depth Oxygen-dependent	Oxygen-dependent Lower efficiency than PDT/PTT Potential cavitation damage	Limited penetration depth Risk of overheating Incomplete ablation
